# Molecular Characterisation of the Nuclear Localisation Sequence Binding Properties of *Toxoplasma gondii* Importin α

**DOI:** 10.3390/cells15201861

**Published:** 2026-10-13

**Authors:** Diksha Mehta, Barnava Banerjee, Fasséli Coulibaly, Kylie M. Wagstaff, Swati Patankar, Prasenjit Bhaumik, David A. Jans

**Affiliations:** 1 Department of Biosciences and Bioengineering, Indian Institute of Technology Bombay, Powai, Mumbai 400076, India; diksha.mehta@iitb.ac.in (D.M.); barnava.banerjee@iitb.ac.in (B.B.); patankar@iitb.ac.in (S.P.); pbhaumik@iitb.ac.in (P.B.); 2 Department of Biochemistry and Molecular Biology, Monash Biomedicine Discovery Institute, Monash University, Monash, Clayton, VIC 3800, Australia; fasseli.coulibaly@monash.edu (F.C.); kylie.wagstaff@monash.edu (K.M.W.)

**Keywords:** *Toxoplasma gondii*, nuclear import, NLS peptides, biophysical assays, MD simulations

## Abstract

The classical nuclear import pathway in eukaryotic cells is mediated by the importin (IMP) α and β1 heterodimer, where IMPα acts as an adaptor protein that recognises and binds to nuclear localisation signals (NLSs) on nuclear protein cargoes. IMPα from *Toxoplasma gondii* (TgIMPα), the causative agent of toxoplasmosis, is an essential gene and validated druggable target, but its NLS binding properties remain largely uncharacterised. To investigate the NLS binding properties of purified TgIMPα we designed NLS peptides and compared its properties with those of mammalian (mouse) IMPα by AlphaScreen binding assays. The well-characterised viral NLS from simian virus SV40 large tumour-antigen (T-ag NLS) was recognised by TgIMPα with low-nM binding affinity comparable to *Mus musculus* IMPα (MmIMPα), whereas a *T. gondii*-specific NLS from the GCN5 gene product (TgGCN5) exhibited high affinity binding to TgIMPα but not MmIMPα. Mutational analysis of basic residues within the NLSs indicated pronounced binding selectivity. To elucidate the structural basis of these interactions, molecular dynamics (MD) simulations of an *ab initio* TgIMPα model bound to each NLS was performed. Key polar, salt-bridge, and cation-π interactions driving NLS recognition were identified. Critically, the lysine at position 2 (Lys128 in T-ag and Lys95 in TgGCN5) of each NLS engages conserved threonine and aspartate residues at the major binding site, playing an essential role in binding stability. Strikingly, TgIMPα displayed a more extensive and distinct cation-π interaction network than MmIMPα, revealing that, while the overall NLS binding architecture is conserved, specific pocket variations underlie differential high affinity recognition. Collectively, this study provides insights for the first time into the molecular distinctions in NLS binding between parasite and mammalian IMPα, potentially representing the foundation for future selective targeted drug design or peptidomimetic strategies against *T. gondii*.

## 1. Introduction

Nuclear protein import mediated by members of the importin (IMP) superfamily of nuclear transporters enables proteins synthesised in the cytoplasm to enter the nucleus for critical roles in processes essential to eukaryotic cells such as transcription, gene regulation, and DNA repair and replication [1,2]. Proteins destined for the nucleus harbour a nuclear localisation signal (NLS), a short stretch of mostly positively charged amino acids [3,4,5], that functions as a targeting signal recognised by the IMPs. In the classical nuclear protein import pathway, a heterodimer complex of IMPα/β1, where IMPα binds IMPβ1 via its N-terminal IMPβ1 binding (IBB) domain, binds the NLS of the nuclear protein import cargo, and docks the ternary complex at the nuclear envelope through interactions of IMPβ1 with the nucleoporin components of the nuclear pore complex. Transport through the pore into the nucleus follows through analogous IMPβ1–nucleoporin interactions, prior to dissociation of the complex within the nucleus upon binding to IMPβ1 of the small monomeric guanine nucleotide binding protein Ran in activated GTP-bound form.

IMPα has three distinct structural domains: the N-terminal IBB domain [6], a central super-helical assembly of ten armadillo (ARM) repeats, and a C-terminal domain responsible for IMPα recycling to the cytoplasm [7]. Each ARM repeat is composed of three α-helices—H1, H2, and H3—connected by interhelical loops [8,9,10]. The structured ARM repeat region contains two NLS binding sites: the major (ARM2–4) and the minor (ARM6–8). These sites are occupied by two different classes of classical NLSs: monopartite NLSs with a single basic cluster that predominantly occupy the major NLS site, and bipartite NLSs that contain two basic clusters separated by a linker and occupy both major and minor NLS binding sites [11,12].

The IBB domain of IMPα contains a positively charged sequence that resembles an NLS [9,13]. In the absence of IMPβ1, this sequence binds to the major NLS binding site within the ARM repeats of IMPα, effectively blocking the binding of nuclear cargoes. This intrinsic regulatory mechanism, which prevents premature or nonspecific cargo binding, is termed ‘autoinhibition’ [8,13,14,15]. IMPα proteins from different organisms show varying levels of autoinhibition [14,16]. Of potential therapeutic importance, the IMPα proteins from the apicomplexan parasites *Toxoplasma gondii* (TgIMPα) and *Plasmodium falciparum* (PfIMPα) exhibit respectively distinctive weak and negligible autoinhibition properties [17,18].

Although autoinhibition of TgIMPα has been described [17,18], the molecular basis of TgIMPα’s NLS binding properties remain unexplored. In this study we employed the highly sensitive AlphaScreen binding assay to compare the ability of TgIMPα to bind to NLS peptides, compared with that of *Mus musculus* IMPα (MmIMPα) for the first time. We utilised two classical monopartite NLSs, including the well-characterised NLS from simian virus SV40 large T-antigen (T-ag NLS), and the only reported *T. gondii*-specific NLS [19]. Although the T-ag NLS was recognised by TgIMPα with low-nanomolar binding affinity comparable to MmIMPα, the Tg-specific NLS from the *T. gondii* GCN5 (general control non-repressed 5) gene product (TgGCN5 NLS) showed high affinity binding to TgIMPα but not MmIMPα. Mutant peptides of the two NLSs probing the role of basic residues in NLS-IMPα interactions indicated binding specificity. To provide mechanistic insight into these observations, structural modelling and molecular dynamics simulations were performed. These analyses revealed a network of stabilising interactions at the NLS–IMPα interface, including polar contacts, salt bridges, and a dynamic ensemble of cation–π interactions between the lysine and arginine residues of the NLSs and tryptophan residues lining the NLS binding groove of IMPα. Notably, TgIMPα exhibited a more extensive and distinct pattern of cation-π interactions compared with MmIMPα, suggesting that, while the overall architecture of the NLS binding pocket is conserved between TgIMPα and its mammalian counterpart, specific variations contribute to differential binding affinity and recognition. These differences likely underlie functional specialisation in apicomplexan nuclear import pathways and highlight the potential for exploiting TgIMPα’s unique binding features in the design of selective peptidomimetic inhibitors targeting *T. gondii*.

## 2. Materials and Methods

### 2.1. Expression and Purification of TgIMPα

The *T. gondii* IMPα (TgIMPα; ToxoDB TGME49_252290) gene was codon-optimised, synthesised, and cloned into the pET28a plasmid (GenScript^®^) to generate a cleavable His_6_-tagged construct. The plasmid was transformed into *Escherichia coli* K12 BL21 (DE3) cells and expressed in LB medium with 50 μg/mL kanamycin. Cultures were induced with 0.5 mM IPTG at OD_600_ ≈ 0.8 and grown overnight at 16 °C. Cells were lysed by sonication in 50 mM Tris–Cl (pH 7.4), 500 mM NaCl, 5% glycerol, and the clarified lysate was purified by Ni–NTA affinity chromatography (Cytiva^®^, Marlborough, MA, USA) using a 75–500 mM imidazole gradient. Eluted fractions were dialysed and further purified by size-exclusion chromatography on a Superdex 200 pg column (Cytiva^®^) equilibrated in 20 mM Tris–Cl (pH 7.4), 100 mM NaCl, 5% glycerol. Protein purity was verified by SDS-PAGE, and concentrations were determined spectrophotometrically before aliquoting and storage at −80 °C.

### 2.2. AlphaScreen Binding Assay

The following biotinylated peptides were purchased from GenScript^®^ (Piscataway, NJ, USA): T-ag NLS peptide: SQHSTPPKKKRKV; T-ag NLSmut peptide: SQHSTPPKTKRKV; TgGCN5 NLS peptide: SRDGDSGQSRKRVKREWTSGGR, and TgGCN5 NLSmut peptide: SRDGDSGQSRAAAAREWTSGGR, where the mutations in the NLSmut peptides are underlined.

Lyophilised NLS peptides were resuspended in autoclaved Milli-Q water according to the manufacturer’s instructions and aliquots stored at −80 °C until further use. TgIMPα (expressed/purified as above) or forms of mammalian IMPα from *M. musculus* purified as previously (Walunj et al., 2022) were compared in AlphaScreen assays performed in quadruplicate in 384-well white opaque plates (PerkinElmer) as previously described [20]. Data analysis was performed using GraphPad Prism 10.4.2^®^, and binding curves generated to determine dissociation constants (K_d_) and Bmax [20,21,22].

### 2.3. Structure Modelling of IMPα Bound to NLS Peptides

Full-length amino acid sequences of IMPα from *M. musculus* (UniProt ID: P52293), *Oryza sativa* (UniProt ID: Q71VM4), and *T. gondii* (ToxoDB accession ID: TGME49_252290) were retrieved from the UniProt [23] and ToxoDB [24] databases and multiple sequence alignment was performed using Clustal Omega [25] with default parameters. To visualise sequence conservation and secondary structure correspondence, the alignment was rendered using ESPript 3.0 [26] with reference to the crystal structure of the ARM repeat region lacking the IBB domain (amino acids 72–497) of MmIMPα bound to the the T-ag NLS (PDB ID: 1EJL) [10]. The secondary structural elements from PDB ID: 1EJL were mapped onto the aligned sequences to highlight conserved ARM repeat motifs and key residues within the NLS binding region.

The structure model of full length TgIMPα (ToxoDB accession ID: TGME49_252290, Uniprot ID: A0A125YID0) was retrieved from the AlphaFold Database [27,28]. Residues 1–79 (the IBB domain) and residues 508–545 (the CAS (exportin-2) binding domain) were excluded from the analysis, and the model of the ARM repeat region (residues 80–507) was extracted for further study. This model was aligned with the crystal structure of the ARM repeat region lacking the IBB domain (residues 73–493) of IMPα from *O. sativa japonica* (rice) (OsIMPα) bound to the T-ag NLS at both major and minor NLS binding sites (PDB ID: 4B8O) [15]. OsIMPα was selected as a homologue template due to its high sequence identity of 57%, and a query coverage of ~99% to TgIMPα in NCBI BLAST against the PDB database. Structural superposition revealed a high degree of similarity between TgIMPα and OsIMPα, with a root mean square deviation (RMSD) of 0.96 Å calculated over the Cα atoms of all the superimposed residues. The TgIMPα model was further refined by superimposing it onto the OsIMPα crystal structure in COOT, and the sidechains of conserved residues were readjusted to prevent steric clashes in the structure [29]. The coordinates of the bound NLS were manually extracted from the reported crystal structure of OsIMPα and fitted into the major NLS binding cleft of the TgIMPα model to prepare the NLS-bound structures. For mammalian system, the crystal structure for the T-ag NLS bound to the ARM region (amino acids 72–497) of MmIMPα (PDB ID: 1EJL) was used. For modelling the TgGCN5 NLS-bound complexes, each residue of the T-ag NLS in the major NLS binding pocket of MmIMPα and TgIMPα was systematically substituted to match the TgGCN5 NLS sequence. Thus, a shorter TgGCN5 sequence was used in preference to the peptide employed in the AlphaScreen binding assays in order to restrict the model to the residues most likely to directly engage the major NLS binding cleft. Using the core proximal TgGCN5 NLS sequence for structural modelling reduced the likelihood of spurious steric clashes or artefactual flexibility that could otherwise compromise RMSD stability and complex integrity over the course of the MD simulations. Resulting structures were evaluated using ERRAT [30] and PROCHECK [31], both displaying high overall quality, with the TgIMPα-TgGCN5 model achieving an ERRAT quality factor of 99.3, and the MmIMPα-TgGCN5 model scoring 98.1, indicating that the residue-substituted complexes were suitable to perform MD simulations [30].

### 2.4. Molecular Dynamics (MD) Simulations of IMPα with NLS Peptides

All structures for MD simulations were prepared using the solution builder module in CHARMM-GUI [32]. The macromolecules were placed in a cubic box of water molecules, ensuring a minimum distance of at least 10 Å between the protein and the box edges [32]. Appropriate counterions (such as Na^+^ or Cl^−^) were added to neutralise the system and achieve the desired physiological ionic strength of 0.15 mol·L^−1^. The system was then energy-minimised to remove any steric clashes or close contacts. The system was subjected to several stages of equilibration, including NVT (constant number of particles, volume and temperature) and NPT (constant number of particles, pressure and temperature) ensembles. During equilibration, temperature and pressure was controlled using a Nose–Hoover thermostat and Parrinello–Rahman barostat [33,34,35], respectively. Non-bonded interactions were calculated, and the particle mesh Ewald (PME) method was used to compute the long-range electrostatic interactions by specifying the proper PME grid size [36,37]. After equilibration, MD simulations for TgIMPα and MmIMPα bound to the T-ag and TgGCN5 NLSs were carried out for 200 ns, with atomic coordinates were captured every 10 ps. Properties of interest, such as root mean square deviation (RMSD), root mean square fluctuation (RMSF) and hydrogen bond interaction, were calculated on the simulation trajectories. Visualisation of trajectories was carried out using VMD, and graphs were plotted using GraphPad Prism^®^. The images were prepared using PyMOL Molecular Graphics System, Version 3.1.6.1, Schrödinger, LLC and Chimera X 1.1.10. [38].

## 3. Results

### 3.1. TgIMPα Can Bind the T-ag NLS Specifically with High Affinity, in Comparable Fashion to Mammalian IMPα

**Figure 1:**
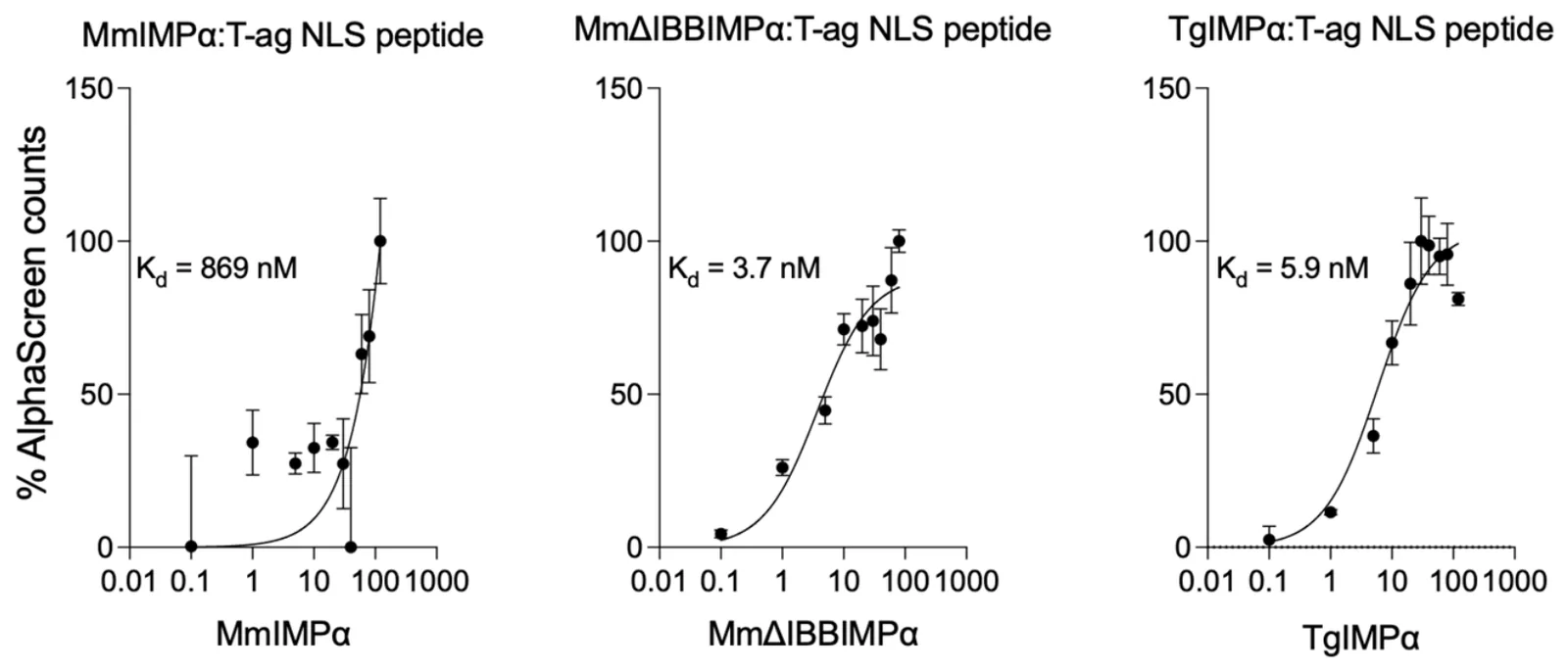
Full-length TgIMPα shows high affinity binding to the T-ag NLS peptide comparable to that of non-autoinhibited mouse IMPα. An AlphaScreen binding assay was used to study the interaction of His_6_-tagged IMPαs with biotinylated T-ag NLS peptide (30 nM). Data represent the mean ± SD from a series of 2–3 independent experiments, with quadruplicate wells in a single experiment (see Table 1 for pooled data).

NLS peptides (see Section 2.2) were designed in order to carry out binding studies to analyse the NLS binding properties of TgIMPα for the first time. The peptides were biotinylated and used in AlphaScreen™ binding assays as previously described [21]. Two forms of MmIMPα were used as controls: full length MmIMPα, which shows autoinhibition and binds NLS-containing proteins with relatively low affinity in the absence of IMPβ1, and the truncated ARM repeat domain form (MmΔIBBIMPα, residues 70–529), which lacks the N-terminal IMPβ1 binding (IBB) domain, and as a result lacks autoinhibition, and can bind NLSs with high affinity. Figure 1 (left and middle panel) compares binding interactions between the T-ag NLS peptide for TgIMPα with both forms of MmIMPα. As expected, full-length MmIMPα exhibited relatively low affinity binding, with a dissociation constant (K_d_) of ~900 nM, in contrast to the high affinity binding (K_d_ of 3 nM) of MmΔIBBIMPα (see Table 1 for pooled data). Strikingly, results for TgIMPα revealed high affinity binding (K_d_ of 5 nM—Figure 1, right panel; Table 1), comparable to those for MmΔIBBIMPα. This documents the ability of TgIMPα to bind a synthetic NLS peptide with high affinity for the first time.

To test binding specificity, assays were performed using a derivative of the T-ag NLS peptide (T-ag NLSmut peptide—see Materials and Methods) where the critical Lys128 residue [39] is substituted with Thr (Supplementary Figure S1). Both full length (left panel) and non-autoinhibited MmIMPα (middle panel) exhibited markedly (60–80%) reduced binding to the NLSMut peptide, relative to the wild-type NLS peptide (Supplementary Figure S1). Similarly, TgIMPα (Supplementary Figure S1, right panel) showed greatly (c. 60%) reduced binding to the NLSMut peptide. These results indicate that TgIMPα, like mouse IMPα, binds the T-ag NLS specifically.

### 3.2. The TgGCN5 NLS Is Recognised Specifically and with High Affinity by TgIMPα but Not MmIMPα

**Figure 2:**
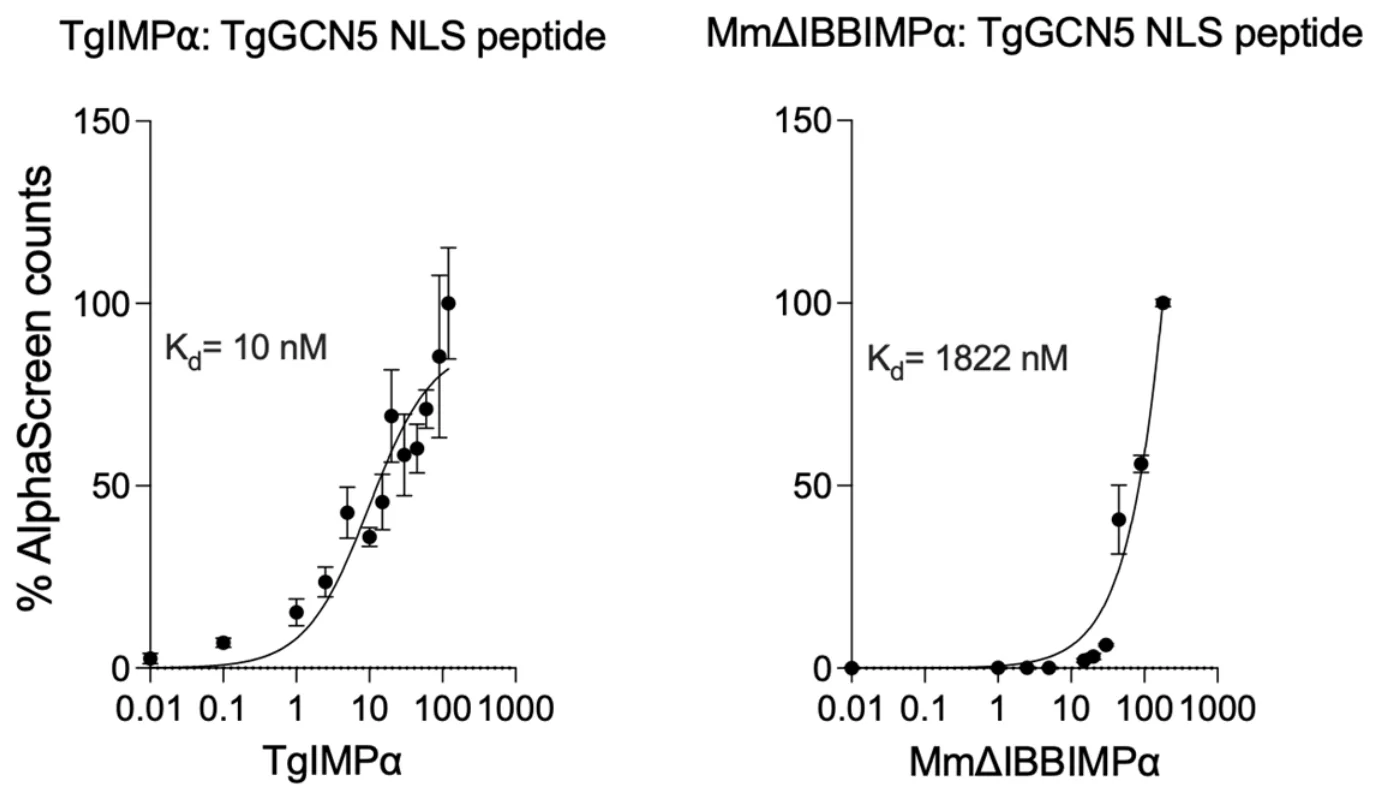
TgIMPα but not MmIMPα binds with high affinity to the TgGCN5 NLS. Alphascreen was performed as per the legend to Figure 1 except that the TgGCN5 NLS peptide was at 80 nM. Data represent the mean ± SD from 3 independent experiments, each performed in quadruplicate (see Table 1 for pooled data).

Only one NLS has thus far been reported for *T. gondii*, that from the transcriptional regulator/histone acetylase GCN5 [19,20], a homolog of yeast GCN5 [40,41]. Both TgIMPα and the MmIMPα/β1 heterodimer have previously been shown to bind with high affinity to a TgGCN5-GFP fusion protein comprising GCN5 residues 1–101 [42], including the RKRVKR^99^ core sequence of the NLS, which has been shown to be essential for nuclear targeting in *T. gondii* tachyzoites [17,19]. We designed a synthetic peptide spanning GCN5 resides 85–106 (SRDGDSGQSRKRVKREWTSGGR^106^) and evaluated its binding to TgIMPα and MmΔIBBIMPα using the AlphaScreen™ assay as above. High affinity (K_d_ ~10 nM) binding was observed for TgIMPα, but MmΔIBBIMPα showed >180-fold lower binding (K_d_: 1822 nM) (Figure 2; see Table 1 for pooled data). Importantly, this is the first time that an NLS sequence from *T. gondii* has been shown to be recognised directly by TgIMPα; the low affinity binding of MmIMPα to the TgGCN5 NLS implies distinct NLS recognition properties in TgIMPα.

Whilst substitutions in classical NLSs are typically limited to a small number of residues, with Thr or Ala substitution of Lys128 well-documented to effect close to a loss-of-function effect [43,44], published analysis with respect to the TgGCN5 NLS is limited to a derivative where the four central residues are substituted by Ala [20]. Specificity of TgGCN5 NLS binding was accordingly tested using a derivative of the TgGCN5 NLS peptide (TgGCN5 NLSmut peptide), where the four central residues were substituted with Ala (SRDGDSGQSRAAAAREWTSGGR). Results from the AlphaScreen assay revealed markedly reduced binding (c. 20% wild type levels) for the NLSmut peptide compared to the wild type peptide (Supplementary Figure S2), consistent with the idea that TgIMPα binds the GCN5 NLS specifically. It is noteworthy that both the T-ag and TgGCN5 NLSmut peptides showed low level residual IMPα binding (Supplementary Figure S2); whether this may be due to contributions to binding of residues flanking the core NLS residues, as observed for other NLSs [45,46], is unclear at this stage.

### 3.3. A Structural Model of TgIMPα and Its NLS Binding Site

The binding studies (Figure 2) indicate differences in the ability of TgIMPα and MmIMPα to recognise the TgGCN5 NLS. To investigate the mechanistic basis of these observations, we wanted to perform a molecular dynamics (MD) simulation study. As no experimental structure exists for TgIMPα, it was decided to generate an AlphaFold-predicted model [27,28] based on the extensive conservation of the ARM repeat fold among IMPαs; an AlphaFold model was chosen to try to reduce bias in subsequent analysis of the NLS binding groove, in contrast to a homology model dependent on the choice of template, which would tend to constrain conformation in the context of template [47]. Our resultant AlphaFold model was supported by its close agreement with the crystal structure of OsIMPα (see Supplementary Figure S3); validation metrics are indicated in Supplementary Figure S3b. This model was used to evaluate similarities in NLS recognition based on the results from Figure 1; TgIMPα was modelled in complex with the T-ag NLS positioned in the major NLS binding cleft. To examine species-specific differences, parallel simulations were carried out using the TgGCN5 NLS. Simulations focused on the major NLS binding site, the predominant site binding classical monopartite NLSs, based on previous observations [10,11,15].

Sequence alignment of MmIMPα, OsIMPα, and TgIMPα (Supplementary Figure S4a—see also pairwise comparisons in Supplementary Table S1) highlighted strong conservation across the ARM repeat domains of all three proteins, including residues known to contribute to NLS binding; conservation of secondary structure assignments from the MmIMPα (72–497) structure is also highlighted (Supplementary Figure S4b).

MD simulations for MmIMPα were performed using the crystal structure of MmIMPα (72–497) (lacking the IBB and C-terminus) bound to the T-ag NLS (PDB ID: 1EJL) [10], with the *O. sativa* IMPα (OsIMPα) structure (residues 73–494, again lacking the IBB and C-terminus; PDB ID: 4B8O), which shares ~63% sequence identity with ARM domain TgIMPα and has been crystallised in complex with the T-ag NLS [15] as an additional structural reference to help model TgIMPα (80–507) binding to the T-ag NLS. In the case of MD simulation of TgGCN5 NLS binding to MmIMPα and TgIMPα, a substitution-based modelling approach was used where the T-ag NLS sequence was systematically changed to that of the TgGCN5 NLS within the major binding pocket of TgIMPα(80–507), based on the MmIMPα (72–497)-T-ag NLS structure (PDB: 1EJL), enabling direct comparison of binding modes between MmIMPα and TgIMPα (see Section 2.3/Section 2.4). All MD simulations/structure validations below were based on these structural models.

Backbone RMSD value monitoring in the simulated complexes relative to the starting structures indicated maintenance of structural stability throughout the simulations for MmIMPα (72–497) and TgIMPα (80–507) (Supplementary Figure S5). Radius of gyration (R_g_) estimations indicated minimal fluctuations, implying that the overall tertiary structure was compact throughout the trajectory (Supplementary Figure S5). Together, the RMSD and R_g_ analyses imply that the complexes of both organisms with the two NLSs are structurally stable over the simulation timescale, providing a robust foundation for subsequent detailed comparative analysis.

### 3.4. MD Simulations Highlight Conserved Electrostatic and Cation-π Contacts Underlie T-ag NLS Binding to the Major NLS Binding Site of MmIMPα and TgIMPα

**Figure 3:**
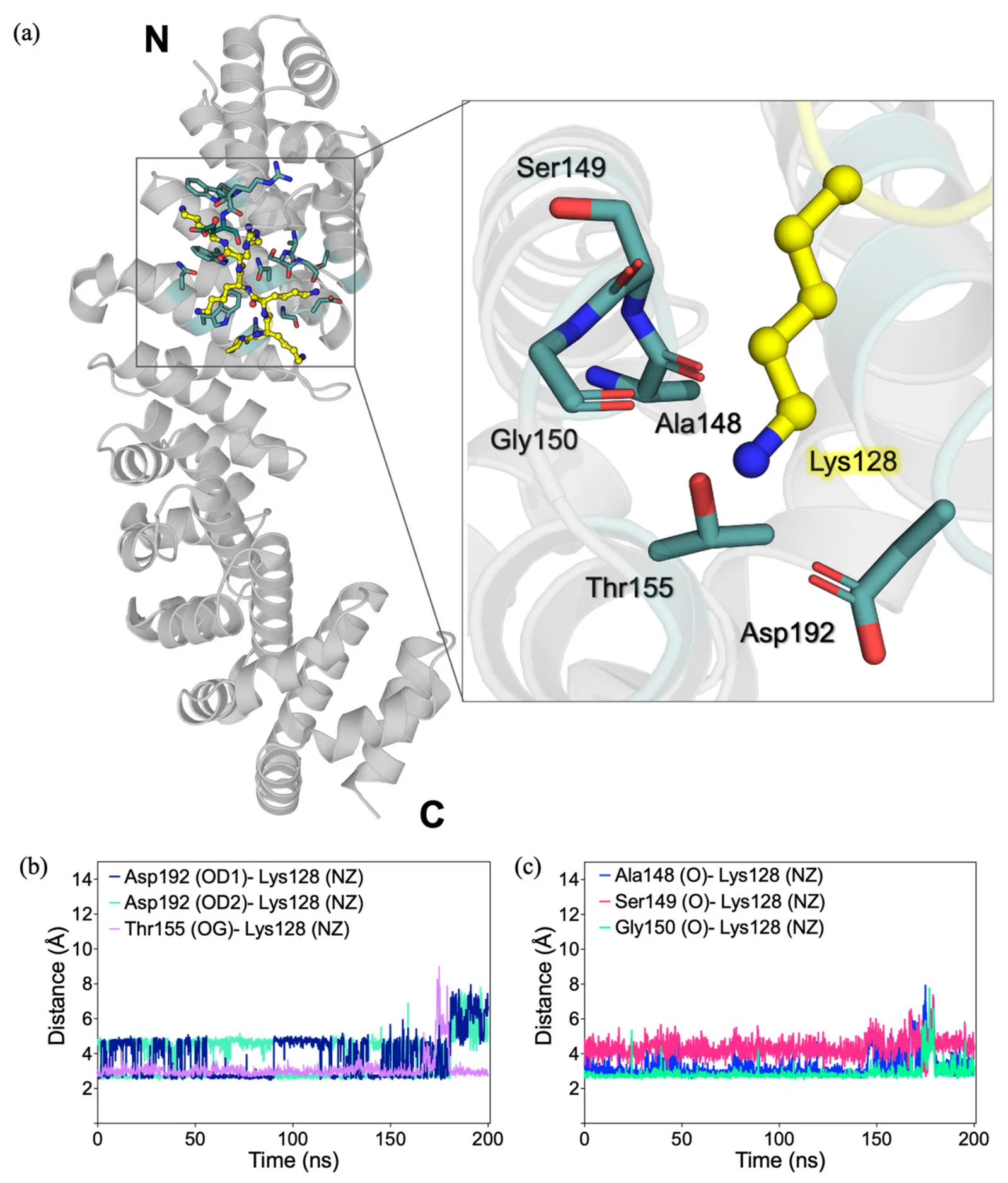
Key interactions of MmIMPα (72–497) with residue (Lys128) of the T-ag-NLS as highlighted by MD simulations. (**a**) Overall structure of T-ag NLS (peptide backbone in yellow) bound to the major NLS binding site of MmIMPα (72–497) (peptide backbone in light blue; N and C represent N- and C-termini respectively); inset: detail of Lys128 in forming a persistent salt bridge with Asp192 and additional polar interactions with Thr155. (**b**) RMSF plots indicate stable interactions between Lys128 and Asp192 and Thr155 with short interatomic distances over the 200 ns simulation run. (**c**) RMSF plots of the main-chain atoms of Ala148, Ser149, and Gly150 with Lys128 of T-ag NLS.

The structural models for MmIMPα (72–497) and OsIMPα (73–494) bound to T-ag NLS reveal the major NLS binding site to be lined with acidic residues that complement the key basic residues of the NLS (see Supplementary Figure S6). To analyse residues involved in NLS binding in more detail, the interatomic distances (<5 Å) were measured between each T-ag NLS residue and the surrounding MmIMPα (72–497) residues in the binding pocket. To assess the stability of these interactions, root mean square fluctuations (RMSFs) were calculated over a 200 ns molecular dynamics simulation, confirming that the interacting residues remained either within polar (<5 Å), salt bridge (<4 Å) or cation-π (3.7–6 Å) interaction distances throughout the simulation [48]. This analysis identified a crucial salt bridge interaction between the ε-amino group (NZ) of Lys128 in the T-ag NLS and the carboxylate oxygen atoms (OD1 and OD2) of Asp192 in the ARM3/ARM4 loop region (Figure 3). Another polar interaction is also evident between the NZ of Lys128 of the T-ag NLS and the side-chain hydroxyl oxygen (OG) of Thr155 in the H3 of ARM2 (Figure 3b). Lys128 is also in close vicinity to the backbone of several residues in the loop region between H2 and H3 in ARM2, which provide an acidic environment to complement the predominantly positive charge of the residues of the NLS (Figure 3c). It is evident that Lys128 of the NLS is involved in multiple interactions with the ARM repeats of the MmIMPα binding cleft, establishing its importance in NLS binding, as complemented by our mutant analysis (Supplementary Figure S1). Beyond the interactions identified in the crystal structure, which implicated Asp192 as the primary binding partner for Lys128 with a possible contribution from Trp231, MD simulations revealed a broader interaction network. These included dynamic contacts between Lys128 and additional polar residues and backbone atoms within the binding pocket.

A few other stable and transient interactions were observed between three Asn (188, 228, 235) residues and Lys127 and Lys129 (Supplementary Figure S7). A transient interaction was observed between the NH_2_ group of Arg130 and the backbone oxygen atoms of Ser105, Arg106, and Glu107 within the ARM1 region of the major NLS binding site, suggesting additional peripheral contacts between the T-ag NLS and ARM1 of MmIMPα (Supplementary Figure S7e,f).

**Figure 4:**
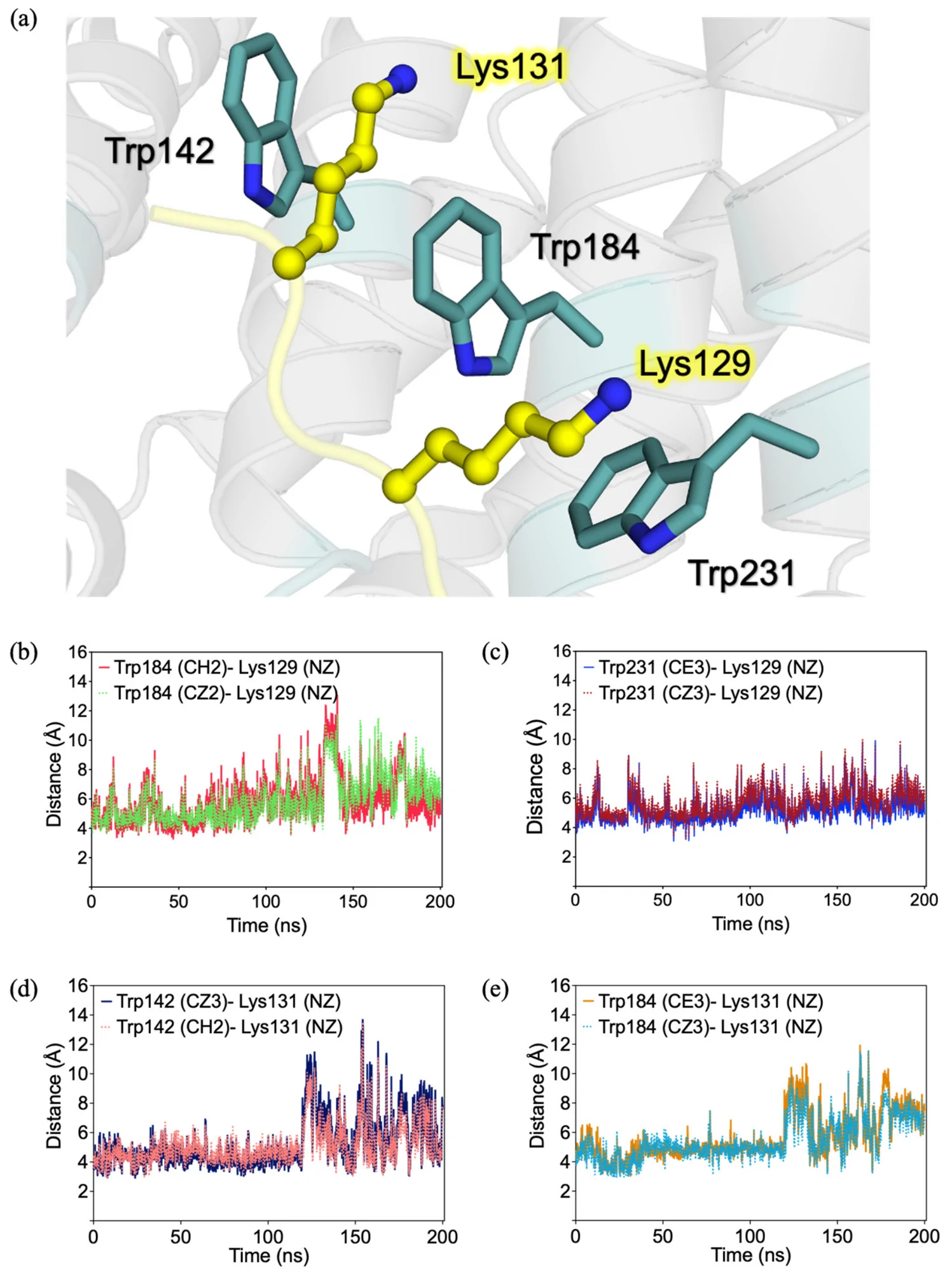
MD simulations highlight formation an aromatic stacking network of Trp142, Trp184, and Trp231 that engages Lys129 and Lys131 through persistent cation-π interactions. (**a**) Ball-and-stick representation shows Trp142, Trp184, and Trp231 of MmIMPα (72–497) (light blue) forming an aromatic stacking network with Lys129 and Lys131 (peptide backbone in yellow) of the T-ag NLS. RMSF plots indicate persistent cation-π interactions between Lys129 and (**b**) Trp184 and (**c**) Trp231, and between Lys131 and (**d**) Trp142 and (**e**) Trp184, over the 200 ns simulation trajectory.

The analysis also highlights a sophisticated network of cation-π interactions that contribute to the stabilisation of NLS binding within the groove. Lys129 and Lys131 of the T-ag NLS form distinct cation-π interactions with Trp residues strategically positioned throughout the binding pocket (Figure 4). These interactions occur when the positively charged Lys/Arg ε-amino groups engage with the delocalised π-electron cloud of the Trp indole ring, generating stabilising noncovalent forces with energetic strengths often comparable to those of hydrogen bonds [49,50]. While cation-π interactions are increasingly recognised as key contributors to molecular recognition in diverse biological systems [51,52], their specific role in IMPα-mediated nuclear import represents an important and underexplored dimension of cargo recognition. Structurally, Lys129 is sandwiched between Trp184 (Figure 4b) and Trp231 (Figure 4c), whereas Lys131 interacts primarily with Trp142 (Figure 4d) and Trp184 (Figure 4e), revealing the strategic architectural arrangement of aromatic residues that reinforces NLS engagement beyond conventional electrostatic complementarity. These findings demonstrate that MmIMPα (72–497) recognises the T-ag NLS through a comprehensive framework of interactions, integrating a central Lys128-mediated electrostatic anchor with distributed cation-π interactions and peripheral contacts that collectively stabilise NLS positioning within the major binding groove.

MD simulations were fully consistent with the published crystal structure (PDB 1EJL) [10], confirming key electrostatic interactions between Lys128-Asp192, Asn188-Lys129, and Asn235-Lys127, all of which maintained shorter interatomic distances throughout the simulation trajectories. Notably, the simulations also identified additional polar and main-chain interactions with Lys128, peripheral contacts with Arg130 and extensive cation-π interactions in the major NLS site.

**Figure 5:**
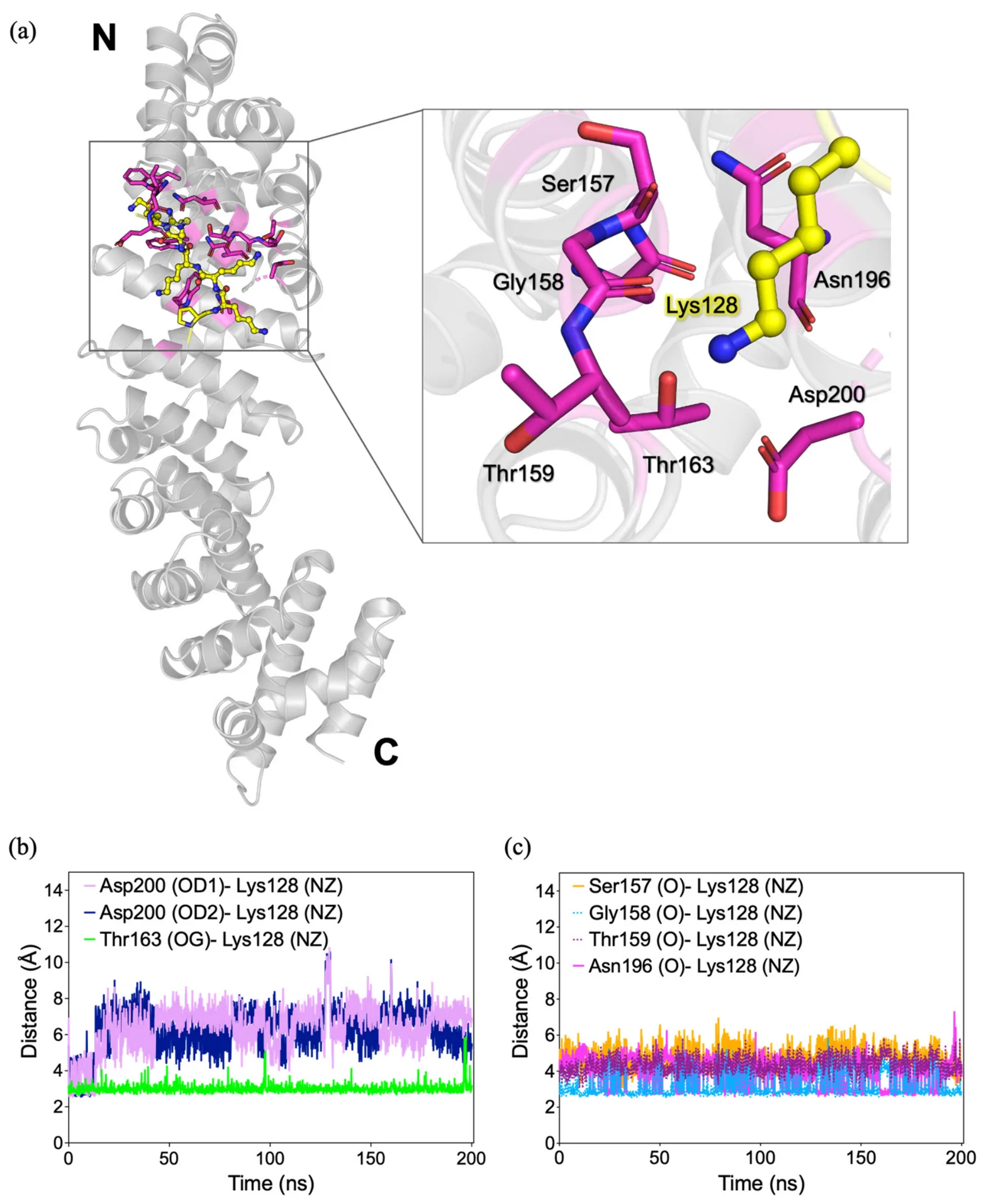
Key interactions between T-ag NLS and TgIMPα (80–507) as highlighted by MD simulations. (**a**) Overall structure of T-ag NLS (peptide backbone in yellow) bound to the major NLS binding site of TgIMPα (80–507) (peptide backbone in pink); inset: zoomed-in view of Lys128 in forming a persistent salt bridge with Asp200 and additional polar interactions with Thr155 and main-chain atoms of Ser157, Gly158, Thr159, and Asn196. (**b**) RMSF plots indicate a persistent salt bridge between the Lys128 side chain and Asp200 and a polar interaction with Thr163. (**c**) RMSF plots between the backbone carbonyl atoms of Ser157, Gly158, Thr159 and Asn196 over the 200 ns simulation trajectory.

To assess the extent of conservation of these NLS binding interactions in TgIMPα, parallel MD simulations were performed assessing TgIMPα (80–507) binding to T-ag NLS. To identify the residues involved in T-ag NLS binding and stabilisation, interatomic distances (<5 Å) were measured between each T-ag NLS residue and the surrounding TgIMPα (80–507) residues in the binding pocket using the modelled structure, as above. At the core of the binding interface, the ε-amino group of Lys128 (NZ) forms a salt bridge with the carboxylate groups (OD1 and OD2) of Asp200 in TgIMPα (80–507) (Figure 5a); but, in contrast to the stable interaction with MmIMPα (72–497) Asp192, the salt bridge appears stable during the early phase of the simulation but appears to slightly weaken over time, as indicated by an increase in the interatomic distances (Figure 5b). In contrast, the polar interaction between the ε-amino group (NZ) of Lys128 and the hydroxyl group of Thr163 (OG) (Figure 5a) remains stable throughout the 200 ns simulation, with interatomic distances consistently ranging between 2 and 4 Å (Figure 5b), mirroring the robust Thr155 interaction in the mammalian system. Structurally, Lys128 is also positioned near the backbone atoms of the loop connecting helices H2 and H3 of ARM2 and shows short interatomic distances with the backbone carbon atoms, recapitulating the spatial arrangement observed in MmIMPα (72–497) (Figure 3c and Figure 5c). Together, the MD simulation data and binding analysis (Supplementary Figure S1) highlight Lys128 as a key residue mediating NLS recognition in both TgIMPα and MmIMPα, despite subtle differences in the dynamics of individual contacts.

**Figure 6:**
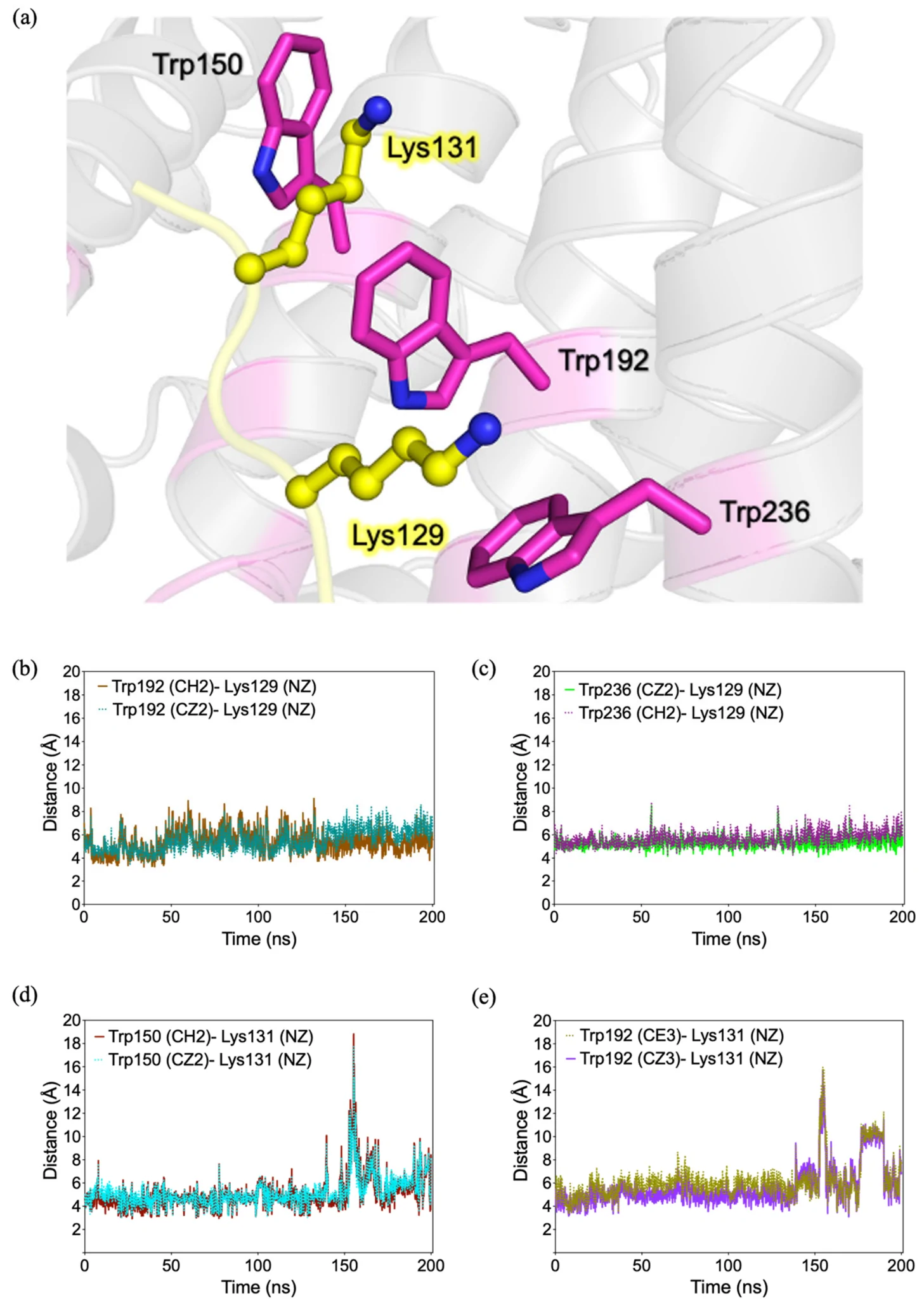
MD simulations highlight cation-π interactions between Lys129 and Lys131 of the T-ag NLS and Trp residues of TgIMPα (80–507). (**a**) Ball-and-stick representation shows Lys129 and Lys131 of the T-ag NLS (peptide backbone in yellow) forming cation-π contacts with Trp150, Trp192, and Trp236 of TgIMPα (80–507) (pink). RMSF plots indicate short interatomic distances between Lys129 and (**b**) Trp192 and (**c**) Trp236, as well as Lys131 and (**d**) Trp150 and (**e**) Trp192, maintained over the 200 ns simulation trajectory.

In addition to the Lys128-mediated contacts, the adjacent NLS residues Lys127, Lys129, and Lys131 engage in stable polar interactions with conserved Asn side chains within the TgIMPα (80–507) binding groove (Supplementary Figure S8). Throughout the 200 ns simulation, the ND2 atom of Asn154 formed a persistent hydrogen bond with the backbone oxygen of Lys131, while Asn196 (OD1) interacted with the backbone nitrogen of Lys129, and Asn240 (ND2) formed a stable contact with the backbone oxygen of Lys127 (Supplementary Figure S8b). Although comparable interactions were observed for MmIMPα (72–497) (see above, Supplementary Figure S7), the corresponding contacts in TgIMPα (80–507) appeared to be more stable across the 200 ns simulation trajectory. Additionally, a transient peripheral interaction was observed between Arg130 (NH1) and backbone carbonyls in the H2-H3 loop of ARM1 (Supplementary Figure S9), providing auxiliary stabilisation outside the major NLS binding site and extending the interaction network beyond the core binding groove. Importantly, cation-π interactions similar to those characterised in MmIMPα (72–497) are also seen for TgIMPα (80–507), where Trp236 and Trp192 form stabilising interactions with Lys129, and Trp192 and Trp150 with Lys131 (Figure 6).

**Figure 7:**
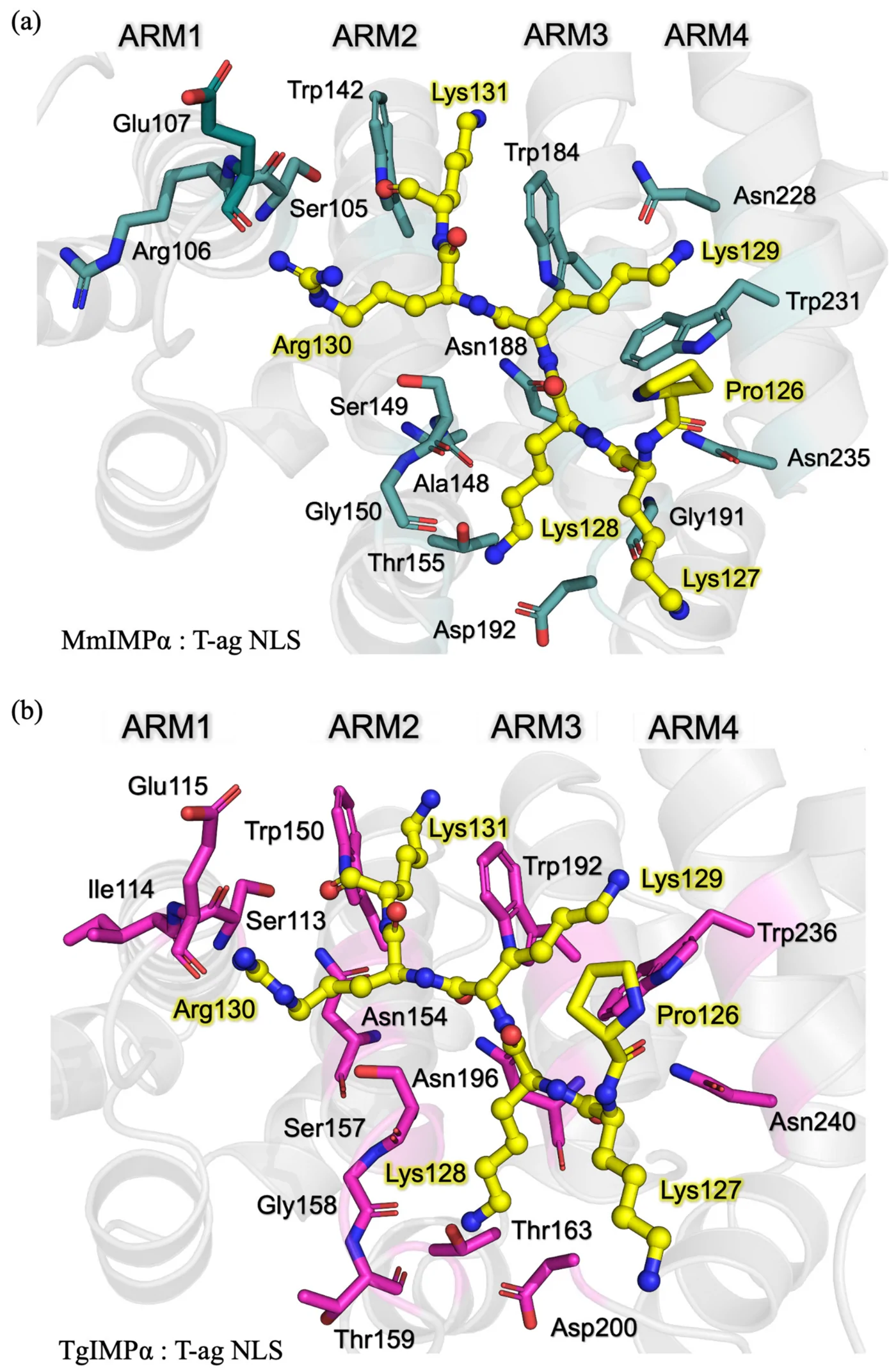
Comparison of binding modes of MmIMPα (72–497) (polypeptide backbone in light blue) (**a**) and TgIMPα (80–507) (polypeptide backbone in pink) (**b**) with the T-ag NLS (peeptide backbone in yellow) as implicated by MD simulations. Ball-and-stick representations are shown, highlighting differences in NLS accommodation within the respective binding pockets. Ball-and-stick representations are shown.

Collectively, the MD results support a multivalent NLS recognition mechanism conserved between TgIMPα and MmIMPα, integrating electrostatic, polar, and aromatic interactions to achieve robust peptide binding, as presented in Figure 7. Additionally, however, differences in the temporal stability of specific contacts, such as the transient Lys128-Asp200 salt bridge in TgIMPα as opposed to the stable Lys128-Asp192 salt bridge in MmIMPα, and the enhanced stability of Asn interactions in TgIMPα compared to MmIMPα, point to subtle variations in binding dynamics that may reflect species-specific adaptations in NLS recognition.

### 3.5. MD Simulation of Binding of the TgGCN5 NLS to TgIMPα Highlights a Combination of Conserved and Unique Molecular Interactions

**Figure 8:**
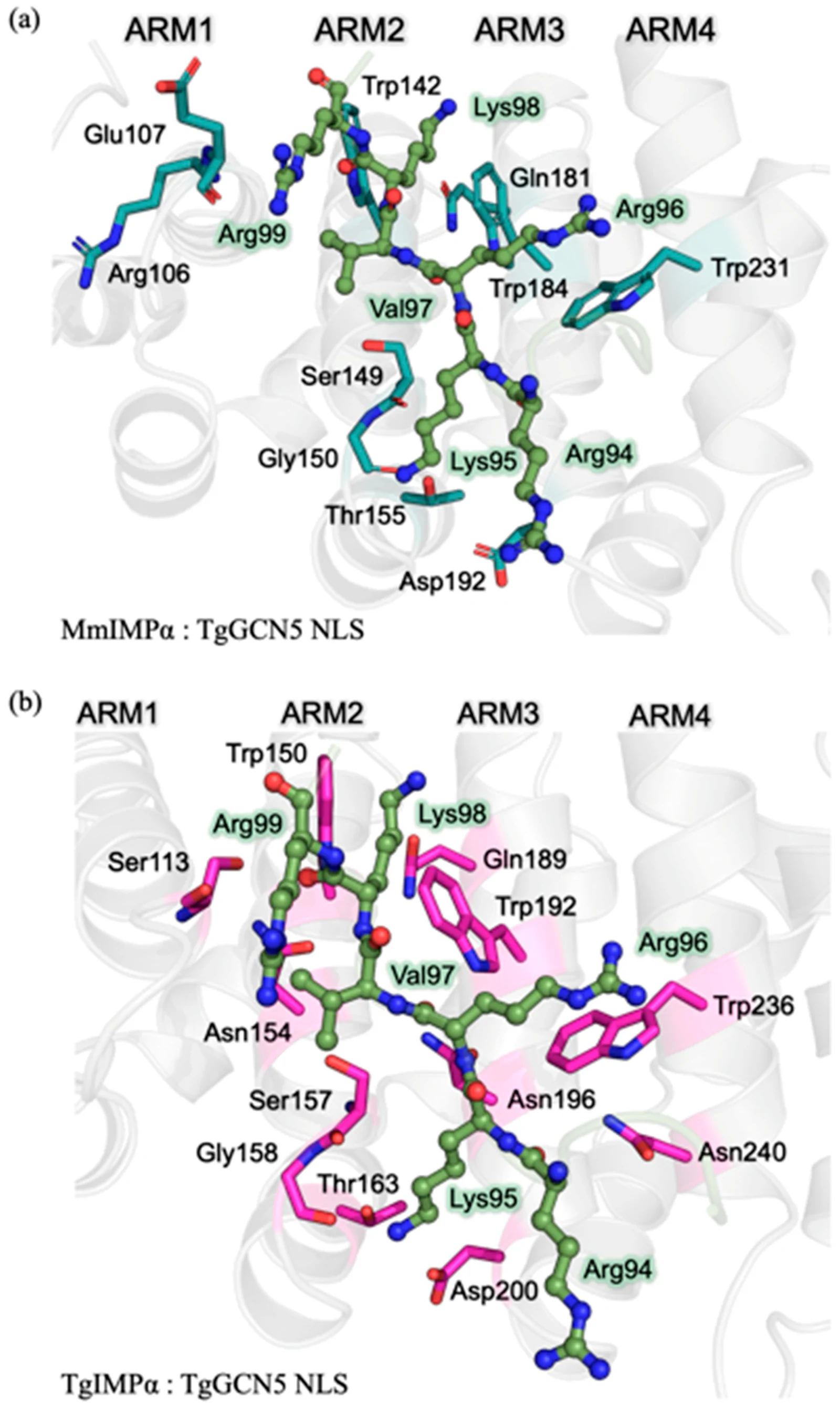
Comparison of binding modes of MmIMPα (72–497) (polypeptide backbone in light blue) (**a**) and TgIMPα (80–507) (polypeptide backbone in pink) (**b**) with the TgGCN5 NLS (peptide backbone in green) as implicated by MD simulations. Ball-and-stick representations are shown, highlighting differences in NLS accommodation within the respective binding pockets.

The TgGCN5 NLS is recognised by TgIMPα with high affinity but MmIMPα with only low affinity (Figure 2), consistent with the idea that TgIMPα, unlike mammalian IMPαs, may have selectivity for NLSs from *T. gondii*. MD simulation RMSD and R_g_ measurements (Supplementary Figure S5) for the TgGCN5 NLS complexes with TgIMPα and MmIMPα confirmed that both complexes were stable over the 200 ns timescale, but detailed interaction analysis revealed distinct binding profiles (Figure 8, Supplementary Figure S6, and see below). Overall, the TgGCN5 NLS formed an extensive network of polar contacts within the TgIMPα binding groove, whereas fewer interactions were observed in the MmIMPα complex. These findings begin to shed light on the apparent selectivity of TgIMPα with respect to *T. gondii* NLSs.

#### 3.5.1. Lys95 Is Central to NLS Binding in the Major NLS Binding Site of IMPα

**Figure 9:**
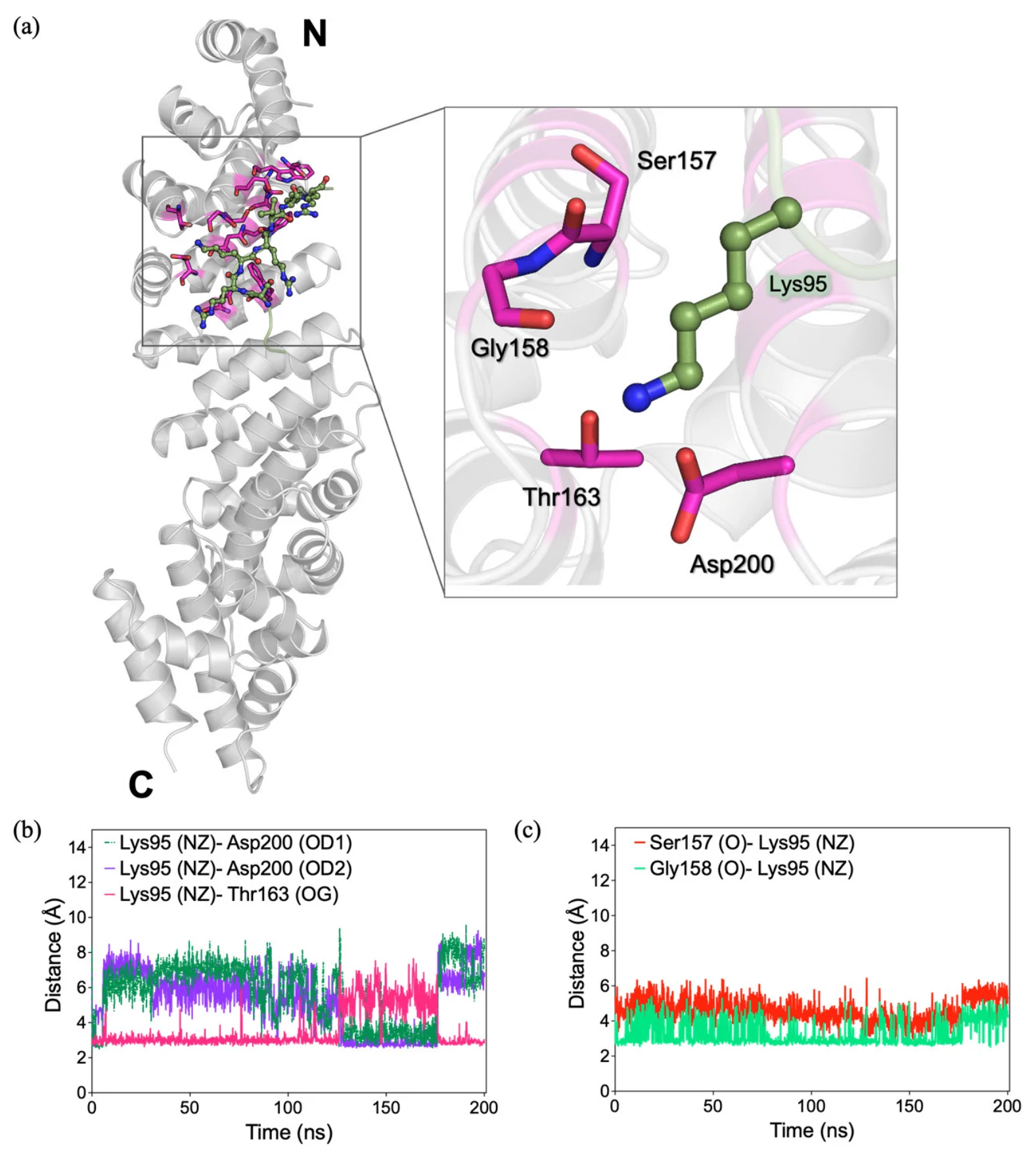
Polar interactions between Lys95 of the TgGCN5 NLS peptide and TgIMPα (80–507) highlighted by the MD simulations. (**a**) Overall structure of the TgGCN5 NLS (peptide backbone in green) bound to the major NLS binding site of TgIMPα (80–507) (polypeptide backbone in pink), with the inset showing a zoomed-in view of the salt bridge interaction between Lys95 of the TgGCN5 NLS and Asp200, a polar interaction with Thr163, and contacts with the backbone carbonyl oxygen atoms of Ser157 and Gly158. (**b**,**c**) RMSF plots indicate variable interatomic distances between Lys95 and Asp200 and Thr163 (**b**) and more persistent interactions with Ser157 and Gly158 (**c**), over the 200 ns simulation trajectory.

In analogous fashion to Lys128 of T-ag NLS, Lys95 of the TgGCN5 NLS appears to interact closely with Asp200 and Thr163 of TgIMPα in our MD simulations (Figure 9). A strong salt bridge between Lys95 (NZ) and Asp200 (OD1/OD2) is observed early in the simulation, then weakens somewhat, restabilises around 120 ns, and then partially disrupts near 176 ns (Figure 9a), indicating a dynamic interaction. The polar contact between Thr163 and Lys95 (NZ) is initially stable, becomes weaker during the run, and later re-establishes (Figure 9a), contrasting with the stable interactions with Lys128 of Asp200 and Thr163 seen for the T-ag NLS (see above). This alternating pattern implies that Lys95 may engage in mutually exclusive interactions with Asp200 and Thr163, highlighting a dynamic more flexible mode of binding of the TgGCN5 NLS within the major groove of the TgIMPα ARM repeat. Additional polar interactions could be visualised between the NZ of Lys95 and the backbone carbonyl oxygen atoms of Ser157 and Gly158 of the loop between H2 and H3 of ARM2 (Figure 9c). Although strongly dependent on Lys95 (Supplementary Figures S1 and S2), these various other contacts/interactions all likely contribute to stabilise TgGCN5 NLS binding to TgIMPα.

#### 3.5.2. Polar Networks and Cation-π Interactions Help Stabilise NLS Binding

Interestingly, transient interactions were observed between Asp200 (OD1/OD2) and the side-chain amino groups (NE, NH1 and NH2) of Arg94 of the NLS (Supplementary Figure S10a–d). Other polar interactions include Lys98 (NZ) with the backbone oxygen atom of Gln189, located in the H2 helix of the ARM3 region, and the backbone nitrogen atom of Lys98 with carbonyl oxygen (OD1) of Asn154 (Supplementary Figure S10e,f). Arg99 engages in multiple stable contacts, with the backbone oxygen of Ser113 forming H-bonds with the three nitrogen atoms (NE, NH1, and NH2) of Arg99 (Supplementary Figure S10g,h).

**Figure 10:**
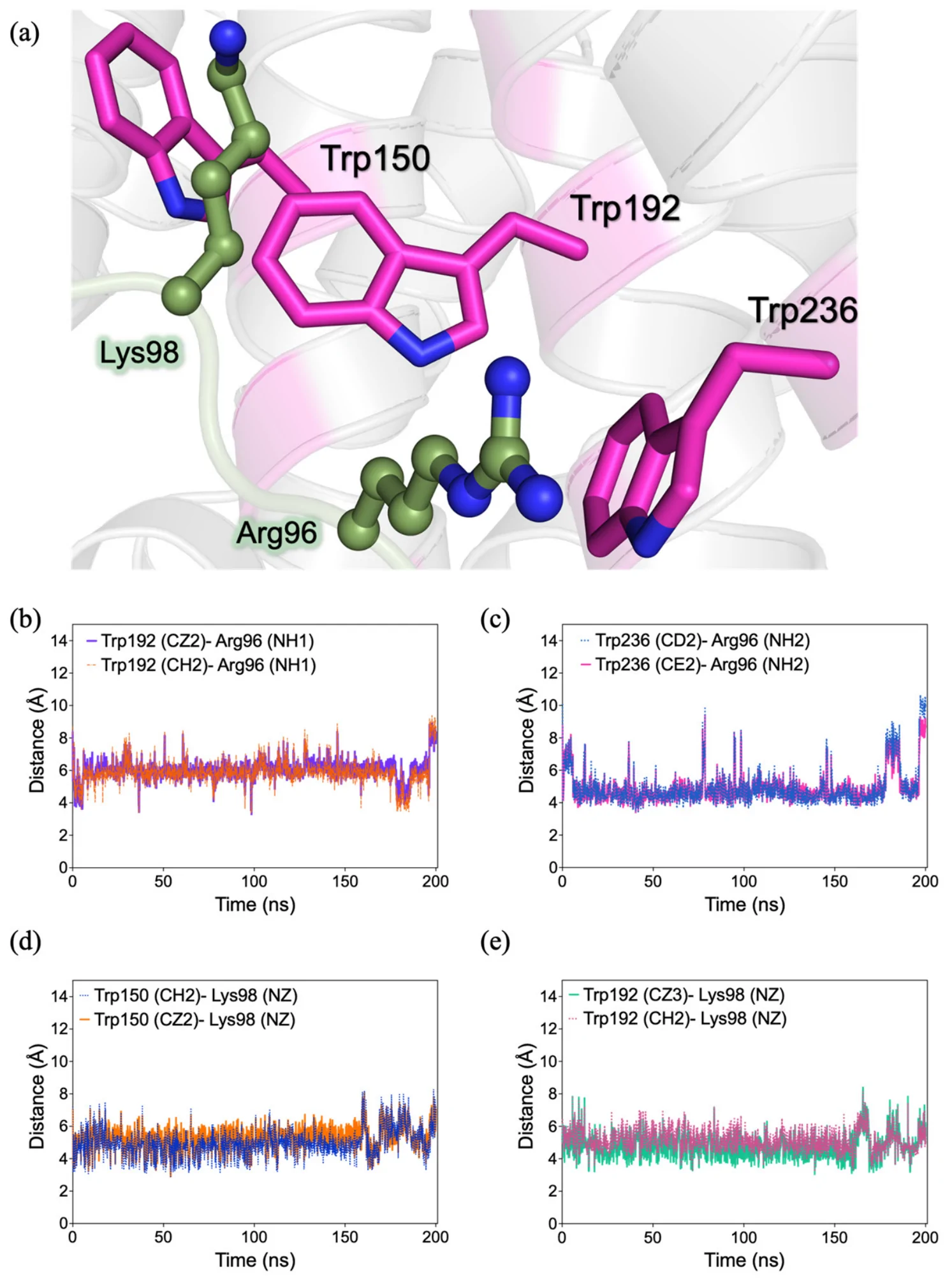
Cation-π interactions between the TgGCN5 NLS (peptide backbone in green) and the major NLS binding groove of TgIMPα (80–507) (pink) highlighted in MD simulations. (**a**) Ball-and-stick representation (top) shows the binding mode of the NZ atom of Lys98 of the TgGCN5 NLS with Trp150 and Trp192, and the guanidinium nitrogen atoms of Arg96 with Trp192 and Trp236 within the major NLS binding groove of TgIMPα (80–507). (**b**–**e**) RMSF curves indicate short interatomic distances between the NH1 atom of Arg96 and Trp192 (**b**), the NH2 atom of Arg96 and Trp236 (**c**), and between Lys98 and Trp150 (**d**) and Trp192 (**e**), over the 200 ns simulation trajectory.

Cation-π interactions have been shown to be a recurring feature in NLS binding interactions with the NLS binding pocket of IMPα [53]; similar interactions are evident in the MD simulation of the TgGCN5 NLS binding to TgIMPα (80–507). Specifically, Arg96 forms stabilising interactions with Trp236 and Trp192, while Lys98 engages Trp192 and Trp150 (Figure 10). The guanidinium group of Arg96 is particularly well-positioned to simultaneously contact both Trp192 and Trp236 (Figure 10). The presence of Arg in this position (compared to Lys in the T-ag NLS—see above) may further enhance the binding stability due to the higher potential of the Arg side chain to form directional contacts with the indole ring Trp [54]. Similarly, the ε-amino group of Lys98 aligns with the indole rings of Trp150 and Trp192 (Figure 10d,e), reinforcing the stability of the peptide binding interface. The involvement of Trp residues in NLS recognition has been noted previously [8,9,10], but the extent to which these cation-π networks may be important for a variety of distinct NLSs has not been characterised in detail; our MD simulations here for the GCN5 NLS give an insight into their potentially broader role in stabilising IMPα-NLS complexes.

In contrast to TgIMPα (80–507), MmIMPα (72–497) showed limited interactions with the TgGCN5 NLS. Conserved contacts included a stable interaction between Lys95 and Asp192 and a polar contact with Thr155, and with backbone carbonyl atoms of Ser149 and Gly150 (Figure S11a–c). Arg99 formed only transient contacts with the backbone oxygen atoms of Arg106 and Glu107, similar to the interaction pattern observed for the T-ag NLS (Figure S11d). Further, the extensive polar interactions observed between MmIMPα and T-ag NLS were absent with TgGCN5 NLS. Stable cation-π interactions were detected between Lys98 and Trp142/Trp184 (Figure S12a–c), and between all three guanidium groups of Arg96 and Trp231 (Figure S12e–g), but not Trp184. An additional transient contact was also observed between Lys98 and Gln181 (Figure S12d). This limited interaction profile is consistent with reduced binding compatibility and aligns with the lower affinity measured in the AlphaScreen assay (Table 1; Figure 2).

As a first step to confirming the role of Trp residues in TgIMPα experimentally, we were able to monitor dose-dependent increases in intrinsic Trp fluorescence of full-length TgIMPα in the absence and presence of NLS peptides (results not shown), consistent with the key involvement of Trp residues in NLS binding by TgIMPα implicated in the MD simulation studies.

Table 2 summarises the residue-level interactions highlighted in the MD simulations comparing the key interactions of MmIMPα with TgIMPα for the T-ag (top section) and GCN5 (bottom shaded section) NLSs. Importantly, the table implies a potentially distinct mode of TgGCN5 NLS recognition by TgIMPα (80–507) compared to MmIMPα (72–497). While MmIMPα shows only a minimal subset of conserved contacts and relies on transient or weak interactions, TgIMPα engages the TgGCN5 NLS through a stable Lys-anchored core supported by an extended network of polar and aromatic interactions. Based on the MD simulations here, it would appear that the T-ag and GCN5 NLSs may exploit the largely conserved binding groove in unique ways, reshaping local interaction networks rather than relying on a single universal binding configuration, likely the basis for the ability of the TgGCN5 NLS to be recognised by TgIMPα but not MmIMPα with high affinity (Figure 2). The implication is that these unique interaction modalities may represent targets for the development of anti-parasitic agents that are selective for *T. gondii*.

## 4. Discussion

This study provides insights into the molecular distinctions in NLS binding between *T. gondii* and mammalian IMPα for the first time. It has previously been shown that NLS binding by IMPα is regulated through an autoinhibitory mechanism dependent on the IBB [9,13,16,17,18,20]; autoinhibition is weaker in apicomplexans including *T. gondii* [17,18], indicating a potential mechanistic difference in nuclear import in apicomplexans compared to mammals, which may be able to be exploited therapeutically. Clearly, a detailed understanding of NLS binding by TgIMPα is central to elucidating mechanistic differences in NLS recognition between the apicomplexan and mammalian systems; our study here uses direct IMPα-NLS peptide binding studies and structural modelling/MD simulations as appropriate to begin to highlight potential differences for the first time.

Our direct binding studies demonstrated specific high affinity binding of both the T-ag NLS and a *T. gondii*-specific NLS (from TgGCN5) by full-length TgIMPα for the first time. In comparison, non-autoinhibited (IBB-truncated) but not full-length mammalian IMPα could recognise the T-ag but not TgGCN5 NLS with high affinity. These results not only confirm the weaker autoinhibition of TgIMPα compared to mammalian IMPα but also imply the existence of species-specific differences in the NLS binding interaction that may determine affinity for particular NLSs.

Previous crystallographic studies have documented the key atomic interactions between NLSs and IBB-truncated mammalian, plant and yeast IMPαs [8,10,15], but no such study has proved possible as yet for an apicomplexan IMPα. This study is the first to generate a *de novo* model of TgIMPα structure, validated on the published IMPα structures, and begin to analyse the molecular basis of TgIMPα-NLS binding in MD simulations; simulations for the T-ag NLS-MmIMPα complex served by way of comparison, underlining the roles of conserved Asp, Asn, and Trp residues in stabilising the major NLS binding groove. Consistent with functional assays, the simulations support the idea of the central role of Lys128 of the T-ag NLS in forming persistent salt bridge and polar contacts in the ARM2 region of MmIMPα, with additional transient interactions from other basic residues of the NLS further contributing to complex stability. The parallel simulations for TgIMPα here show a conserved interaction profile, reinforcing Lys128’s key role in engaging the NLS binding groove, as well as flag additional specific interactions such as contacts between Asn residues in TgIMPα and Lys131 of the T-ag NLS (see Table 2, top section), which may represent an additional layer of specificity.

**Figure 11:**
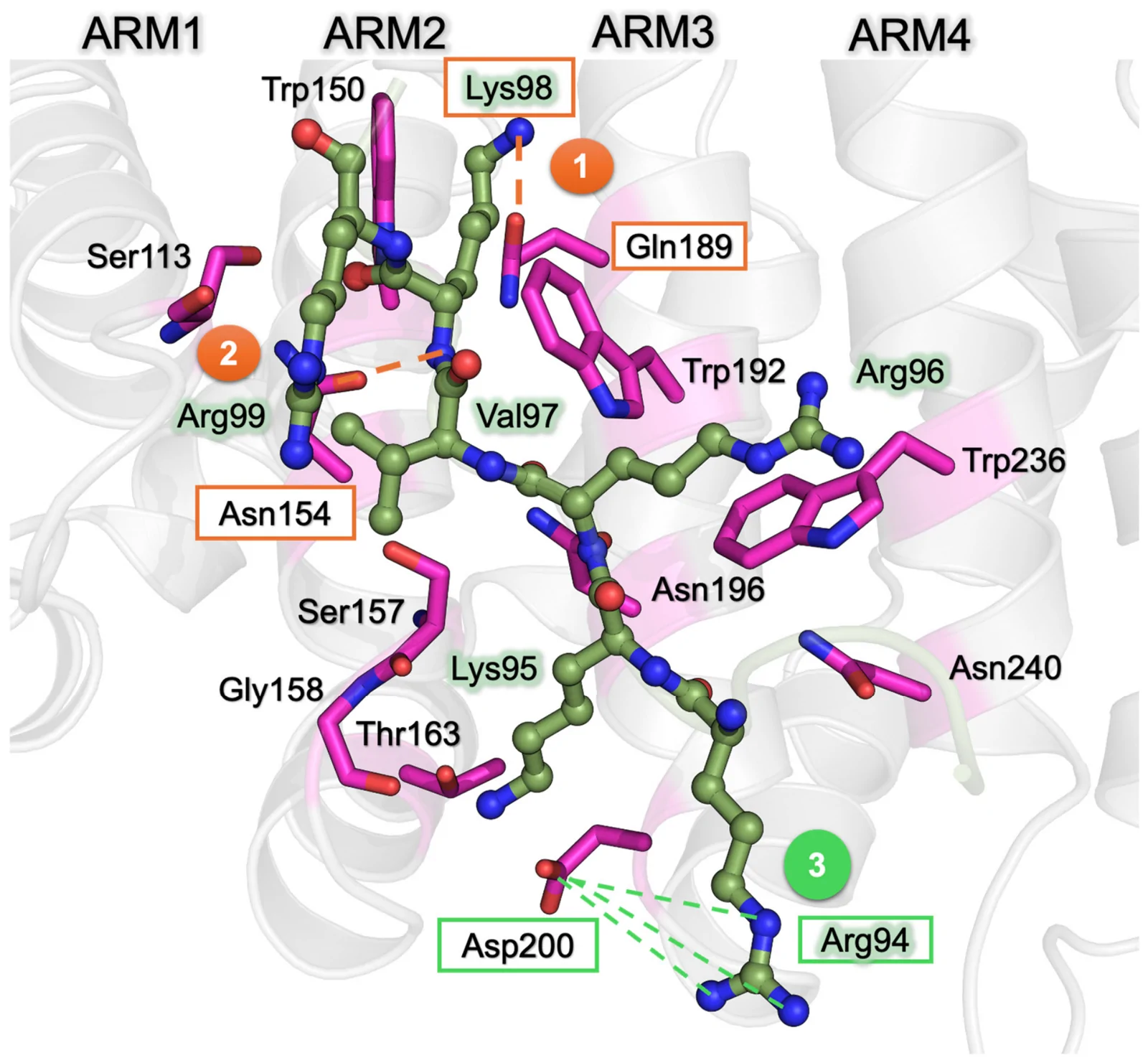
Schematic representation of Tg-specific interactions between TgIMPα (pink) and the TgGCN5 NLS (peptide backbone in green). The ball-and-stick representation is based on Figure 8a and Table 2 and highlights polar interactions (dotted orange lines) between Lys98 and Gln189 and Asn154 (1 and 2 respectively), and the salt bridge interactions (dotted green lines) between the NE, NH1 and NH2 atoms of Arg94 with the carboxylate oxygens of Asp200 (3).

Consistent with full-length TgIMPα but not non-autoinhibited MmIMPα recognising the TgGCN5 NLS with high affinity in direct binding studies, MD simulations for TgGCN5 NLS binding implied more specific interactions for TgIMPα, withTgGCN5 NLS recognition anchored by a conserved lysine-dependent binding core comparable to the T-ag NLS, with Lys95 forming key contacts with Asp200 and Thr163. Interestingly, these contacts displayed greater dynamism in TgIMPα, and were supported by auxiliary polar and transient interactions involving Ser157, Gly158, Lys98, and Arg99, as well as additional cation-π interactions with conserved Trp residues within the binding groove (see Table 2, bottom shaded section). Table 2 summarises T-ag and TgGCN5 NLS binding recognition modes, both those shared between *T. gondii* and mammalian IMPα and those unique to one or the other. The key differences, in terms of binding modes used by TgIMPα and not present in mammalian IMPα to bind the TgGCN5 NLS appear to relate to the polar interactions of Gln189 and Asn154 with Lys98 of the NLS, and (see Figure 11), and the salt bridge interactions of Asp200 with Arg94 of the NLS.

It should be clearly acknowledged that while our MD simulations here provide a structural rationale for observed differences in TgGCN5 NLS recognition by TgIMPα compared to MmIMPα, the specific contributions of individual residues as highlighted above and in Figure 11 are inferred from computational modelling and not experimentally validated. An urgent priority in pursuing this work further will be to perform detailed site-directed mutagenesis of the residues implicated in specific binding of TgIMPα to the TgGCN5 NLS followed by AlphaScreen or comparable binding assays using wild-type and mutant TgGCN5-NLS peptides to confirm effects on binding affinity. Substitution of subsets of the core residues of the NLS as well as flanking residues could also be performed to assess the extent to which individual residues may contribute to the high affinity TgIMPα:TgGCN5-NLS interaction. Experimental confirmation of the proposed roles of the residues in NLS binding specificity will be an essential step to substantiate the mechanistic model proposed here, which, if validated, may represent the basis to pursue targeted therapeutics in the future.

Available strategies to exploit the results in Table 2/ Figure 11 towards inhibitors with specificity for parasite rather than mammalian IMPα-NLS interactions include using *in silico* high throughput screening (HTS) approaches (see [55,56]). Such approaches have been used successfully to identify inhibitors of recognition of the Venezuelan Equine Encephalitis Virus (VEEV) capsid protein by mammalian IMPα, with a number of compounds found with greater than 2-fold efficacy towards the VEEV C-protein as compared with T-ag in ALPHAscreen binding experiments [55]. The latter study also highlighted that fact that structure-based screening alone does not guarantee selectivity or favourable drug-like properties; rather, successive, iterative rounds of *in silico* HTS, experimental measurements, counter-screening and MD simulations to progress appropriate chemical scaffolds to achieve selectivity [56]. In this context, the work here can be seen as part of the initial iterations towards such an important goal.

In conclusion, this study is the first to begin to analyse the molecular basis of specificity of NLS recognition by IMPα from the apicomplexan *T. gondii*, using a combination of direct binding studies, *de novo* modelling, and MD simulation of IMPα-NLS binding interactions. Although the results here require extensive confirmation with direct experimental approaches (see above), this study serves as an important framework towards apicomplexan specific agents targeted at IMPα’s critical functions. The latter is an important focus of future work in this laboratory.

## Figures and Tables

**Table 1:** Summary of K_d_ values for IMPαs binding to NLS peptides.

IMPα: NLS Peptide	K_d_ (nM)
TgIMPα: T-ag NLS peptide *	5.5 ± 0.7
MmΔIBBIMPα: T-ag NLS peptide *	2.8 ± 0.9
MmIMPα: T-ag NLS peptide ^#^	882 ± 19
TgIMPα: TgGCN5 NLS peptide *	10 ± 1.8
MmΔIBBIMPα: TgGCN5 NLS peptide *	>1800

^#^ Results represent the mean ± SD (n = 2) from analysis as per Figure 1, analysed previously in numerous studies, see [20,21]. * Results represent mean ± SD (n = 3) from analysis as per Figure 1.

**Table 2:** Summary of residue-level interactions * observed in 200 ns MD simulations of TgIMPα and MmIMPα bound to the T-ag or TgGCN5 NLS peptides.

NLS	NLS Residue	MmIMPα Residue (s)	TgIMPα Residue (s)	Interaction Type
T-ag NLS	Lys127	Gly191	Asn240	Polar (main-chain)
		Asn235	-	Polar
	Lys128	Asp192	Asp200	Salt bridge
		Thr155	Thr163	Polar
		Ala148	Ser157	Polar (main-chain)
		Ser149	Gly158	Polar (main-chain)
		Gly150	Thr159	Polar (main-chain)
		-	Asn196	Polar (main-chain)
	Lys129	Asn188	Asn196	Polar (main-chain)
		Asn228	-	Polar
		Trp184	Trp192	Cation-π
		Trp231	Trp236	Cation-π
	Arg130	Ser 105	Ser113	Polar (main-chain)
		Arg 106	Ile114	Polar (main-chain)
		Glu107	Glu115	Polar (main-chain)
	Lys131	Trp142	Trp150	Cation-π
		Trp184	Trp192	Cation-π
		-	Asn154	Polar (main-chain)
TgGCN5 NLS	Arg94	-	Asp200	Polar
	Lys95	Asp192	Asp200	Salt bridge
		Thr155	Thr163	Polar
		Ser149	Ser157	Polar (main-chain)
		Gly150	Gly158	Polar (main-chain)
	Arg96	Trp231	Trp236	Cation-π
	Lys98	-	Asn154	Polar (main-chain)
		-	Gln189	Polar
		Trp142	Trp150	Cation-π
		Trp184	Trp192	Cation-π
	Arg99	Arg106	Ser113	Polar (main-chain)
		Glu107	-	Polar (main-chain)

* Residue-level interactions identified across 200 ns MD simulations for TgIMPα and MmIMPα in complex with the T-ag or TgGCN5 NLS peptides. Interactions are attributed on the basis of interatomic distances and RMSF profiles extracted from simulation trajectories and classified as polar, salt bridge or cation-π contacts at the major NLS binding grooves.

## Data Availability

All data relating to this paper can be accessed by corresponding directly with the authors.

## Supplementary Materials

The following supporting information can be downloaded at https://www.mdpi.com/article/10.3390/cells15201861/s1, Supplementary Figure S1. Specificity of T-ag NLS binding by TgIMPα and mouse IMPα. Supplementary Figure S2. Specificity of GCN5 NLS binding by TgIMPα. Supplementary Figure S3. Structural components AlphaFold of TgIMPα. Supplementary Figure S4. Sequence and structural alignment of ARM repeat regions of MmIMPα, OsIMPα and TgIMPα. Supplementary Figure S5. Conformational stability of MmIMPα (72–497) in complex with the T-ag. Supplementary Figure S6. Electrostatic surface representation of the ARM repeats region of MmIMPα (72–497) and TgIMPα (80–507) highlights the acidic nature of the NLS binding groove. Supplementary Table S1. Pairwise comparison of IMPα sequences from *M. musculus*, *O. sativa*, and *T. gondii* together with RMSD measurements. Supplementary Figure S7. Interactions of MmIMPα (72–497) with residues Lys127, Lys129, and Arg130 of the T-ag-NLS as revealed by MD simulations, revealing stable polar interaction networks with conserved Asn residues and backbone oxygen atoms of MmIMPα. Supplementary Figure S8. MD simulations reveal Asn-mediated polar interactions between the T-ag NLS and TgIMPα (80–507). Supplementary Figure S9. MD simulations reveal peripheral interactions between Arg130 of the T-ag NLS and backbone carbonyl oxygens in ARM1 of TgIMPα (80–507). Supplementary Figure S10. MD simulations identify key stabilizing interactions between residues Arg94 and Lys98, and peripheral interactions with Arg99 of the TgGCN5 NLS and binding groove residues of TgIMPα (80–507) over a 200-ns simulation trajectory. Supplementary Figure S11. MD simulations reveal polar and peripheral interactions of the TgGCN5 NLS with MmIMPα (72–497). Supplementary Figure S12. MD simulations highlight cation-π interactions between the TgGCN5 NLS and MmIMPα (72–497).
