# Supplementary material for: Molecular Characterisation of the Nuclear Localisation Sequence Binding Properties of Toxoplasma gondii Importin α
Source: Cells. 2026 Oct 13;15(20):1861. doi: 10.3390/cells15201861 (PMC13702672; doi:10.3390/cells15201861)
Supplement: Supplementary file 1 [file cells-15-01861-s001.zip › cells-4576862-supplementary.pdf]

Supplementary Material

Molecular Characterisation of the Nuclear Localisation Sequence Binding Properties of *Toxoplasma gondii* Importin α

Diksha Mehta [1,2], Barnava Banerjee [1], Fasséli Coulibaly [2], Kylie M. Wagstaff [2], Swati Patankar [1], Prasenjit Bhaumik [1] and David A. Jans [2]

1. Department of Biosciences and Bioengineering, Indian Institute of Technology Bombay, Powai, Mumbai 400076, India

2. Department of Biochemistry and Molecular Biology, Monash Biomedicine Discovery Institute, Monash University, Monash, Clayton, VIC 3800, Australia

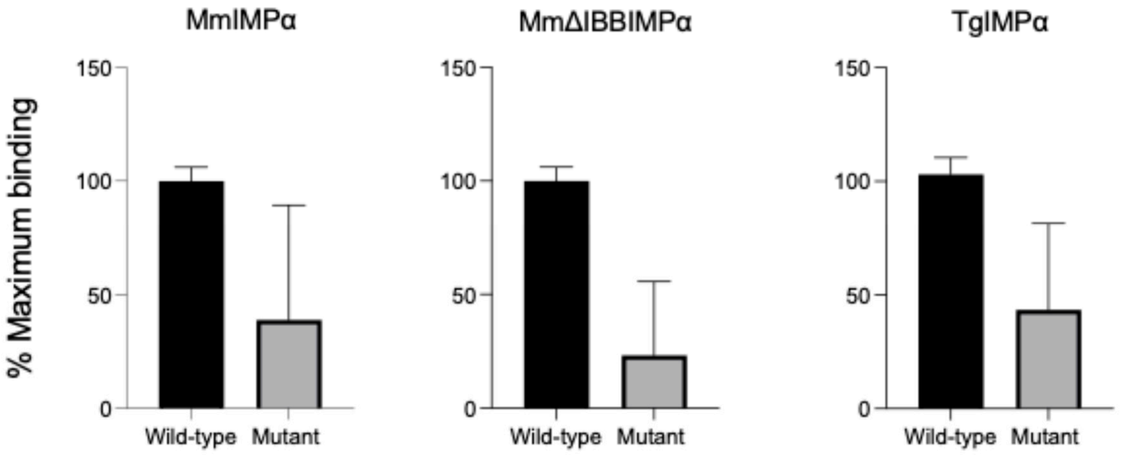

Figure S1. Specificity of T-ag NLS binding by TgIMPα and mouse IMPα. Maximal binding (Bmax) for the wild-type and mutant T-ag NLS peptides was determined by AlphaScreen, performed as per Figure 1. Data is presented as a percentage relative to wild type, where results represent the mean ± SD from 3 independent experiments in quadruplicate in the case of MmΔIBBIMPα and TgIMPα, but 2 independent experiments in quadruplicate in the case of full length MmIMPα. Results for MmIMPα–wild-type and mutant T-ag NLS peptides concur with a number of other studies [20,21].

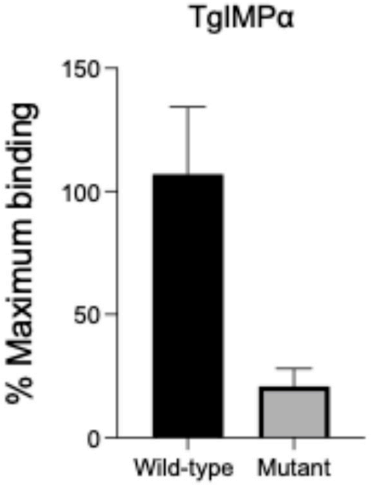

Figure S2. Specificity of GCN5 NLS binding by TgIMPα. Maximal binding (Bmax) for the wild-type and mutant TgGCN5 NLS peptides was determined by AlphaScreen, performed as per Figure 2. Results represent the mean ± SD from three independent experiments, each performed in quadruplicate, where data is presented as a percentage relative to wild type.

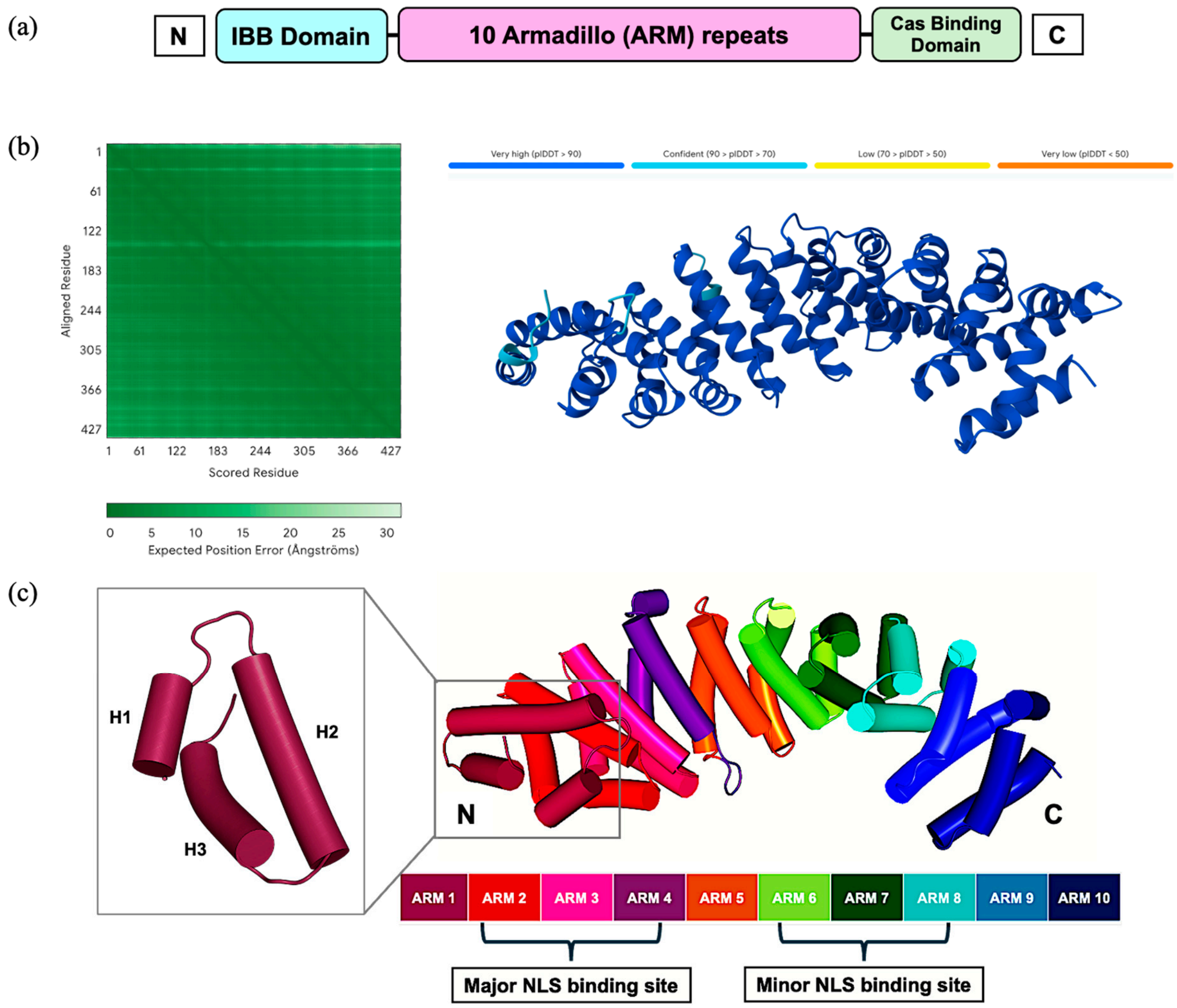

Figure S3. Structural components AlphaFold of TgIMPα. (a) Linear schematic diagram of full-length TgIMPα showing its three structural domains. (b) Snapshots from AlphaFold server with low PAE (predicted aligned error) and high pLDDT scores in the ARM repeat region. (c) Cartoon representation of the 10 ARM repeats showing the overall structural fold. Each ARM repeat is highlighted with a different colour. Linear schematic highlights the major and the minor NLS binding pockets. Inset: Magnified view of ARM repeat 1, highlighting the three helices (H1, H2 and H3) that comprise each repeat.

(a)

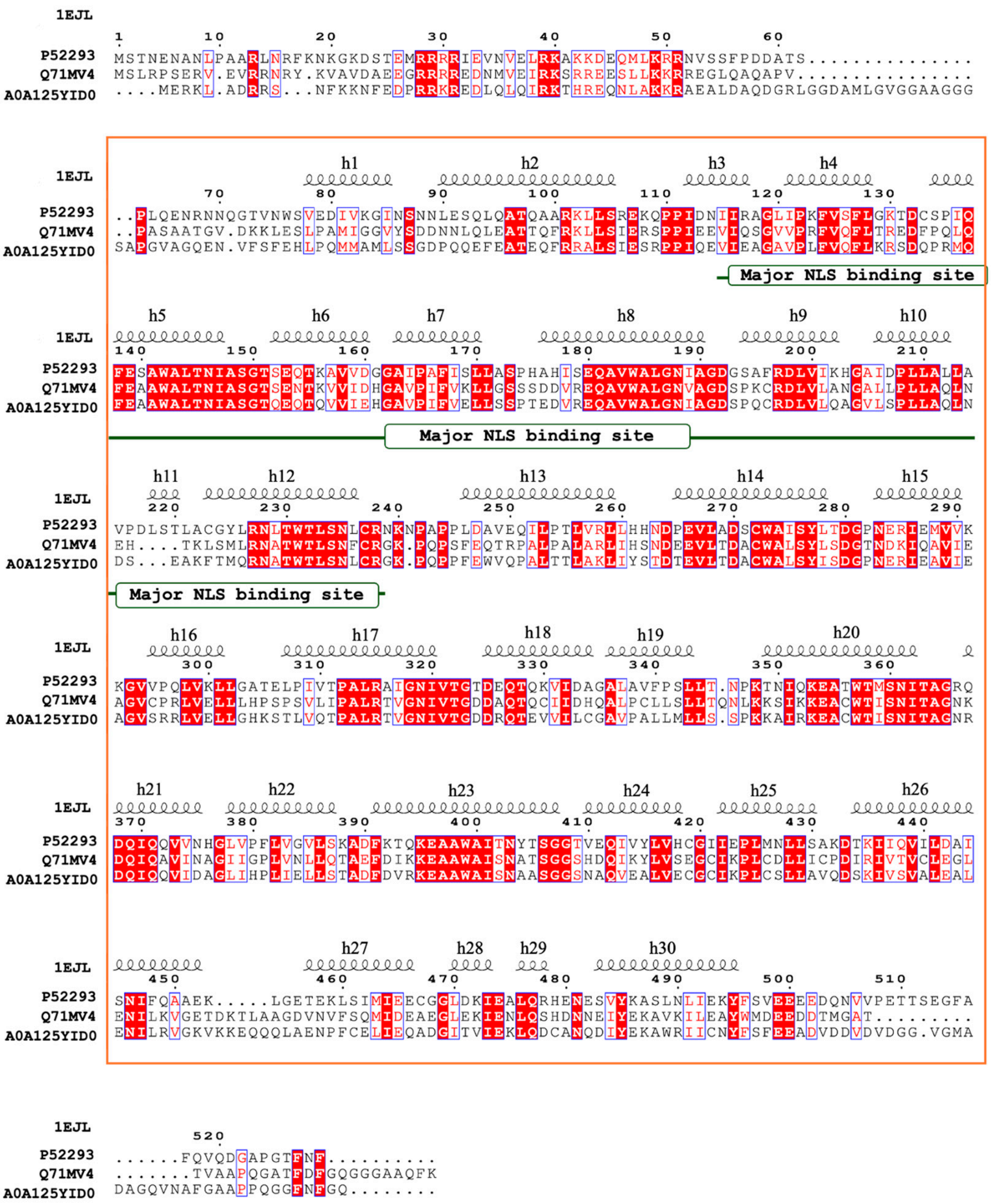
1EJL
P52293
Q71MV4
A0A125YID0
Major NLS binding site
Major NLS binding site
Major NLS binding site

(b)

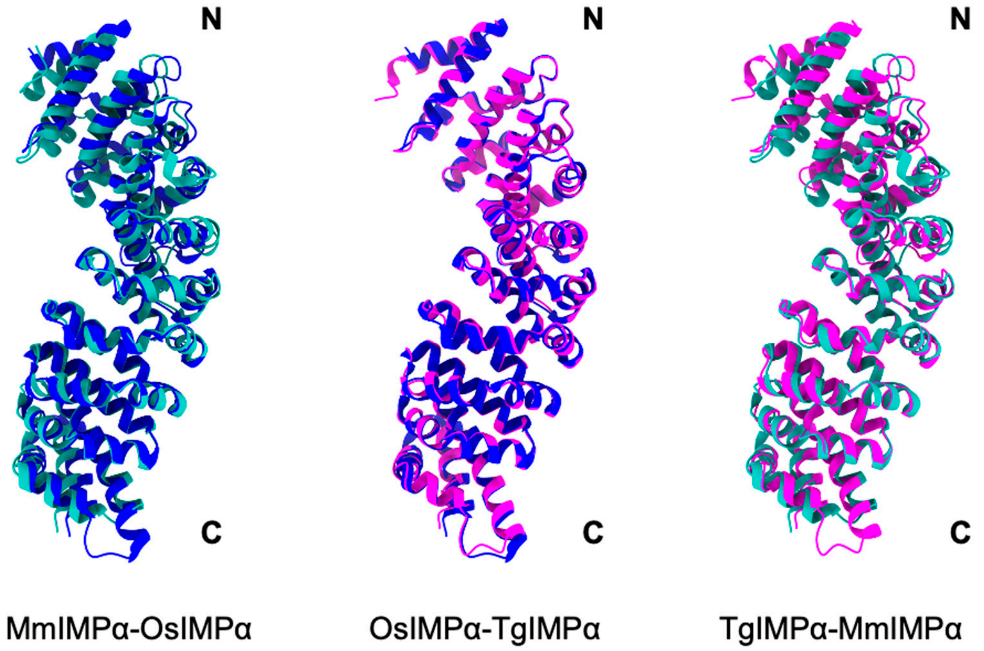
N
N
N
C
C
C
MmIMPα-OsIMPα
OsIMPα-TgIMPα
TgIMPα-MmIMPα

Figure S4. Sequence and structural alignment of ARM repeat regions of MmIMPα, OsIMPα and TgIMPα. (a) Multiple sequence alignment of full-length IMPα proteins from *M. musculus* (P52293/Kpna2/Rch1), *O. sativa* (Q71VM4), and *T. gondii* (A0A125YID0). Numbering above the sequences is for MmIMPα. Regions of helices from the MmIMPα-T-ag NLS structure (PDB: 1EJL) are shown mapped in linear fashion onto the alignment (denoted by h), highlighting secondary structural conservation of the ARM-repeat architecture (orange box) and residues. Residues identical in all three sequences are blocked in red, while residues that are similar across all there are blocked in blue. **(b)** Structural superposition of the structured ARM-repeat domains of MmIMPα(72-497) (cyan), TgIMPα(80-507) (pink), and OsIMPα(74-494) (blue) in ribbon/cartoon form showing overall conservation of fold topology and local variation among species, generated using ChimeraX-1.10.1.

Table S1. Pairwise comparison of IMPα sequences from *M. musculus*, *O. sativa*, and *T. gondii* together with RMSD measurements using PyMOL Molecular graphics software from backbone Cα as per Supplementary Figure S4b.

		**% sequence similarity (identity)[X]**		
Protein		MmIMPα (P52293) (*M. musculus)*	TgIMPα (A0A125YID0) (*T. gondii*)	OsIMPα (Q71VM4) (*O. sativa japonica*)
Full length protein	MmIMPα(1-529)	NA*	59.9 (41.9)	61.8 (45)
	OsIMPα(1-526)	61.8 (45)	67.9 (55.1)	NA*
ARM repeat region	MmIMPα(72-497)	NA*	66.7 (48)	66.3 (49.5)
	OsIMPα(73-494)	66.3 (49.5)	76.2 (63.2)	NA*
RMSD (in Å)	MmIMPα(72-497)	NA*	1.354	1.607
	OsIMPα(73-494)	1.607	0.563	NA*

[X]% sequence similarity is shown (% sequence identity in parentheses) and backbone RMSD values calculated from structural alignment of the ARM-repeat regions.

*Not Applicable

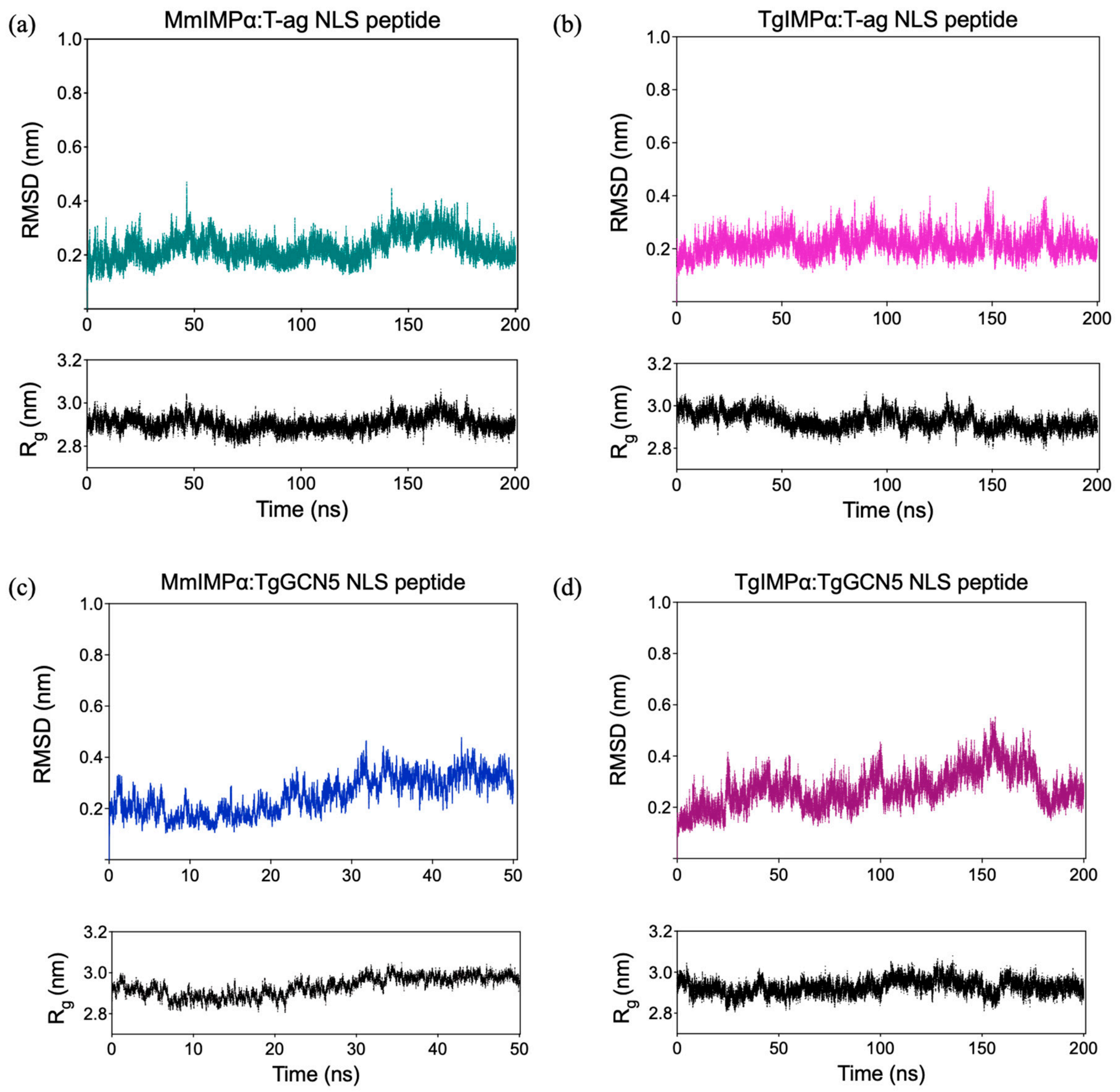

Figure S5. Conformational stability of MmIMPα(72-497) in complex with the T-ag NLS (a) and TgGCN5 NLS (c), and TgIMPα(80-507) in complex with the T-ag NLS (b) and TgGCN5 NLS (d) over 200 ns MD simulations, assessed by backbone RMSD (upper panels) and radius of gyration (R_g) (lower panels).

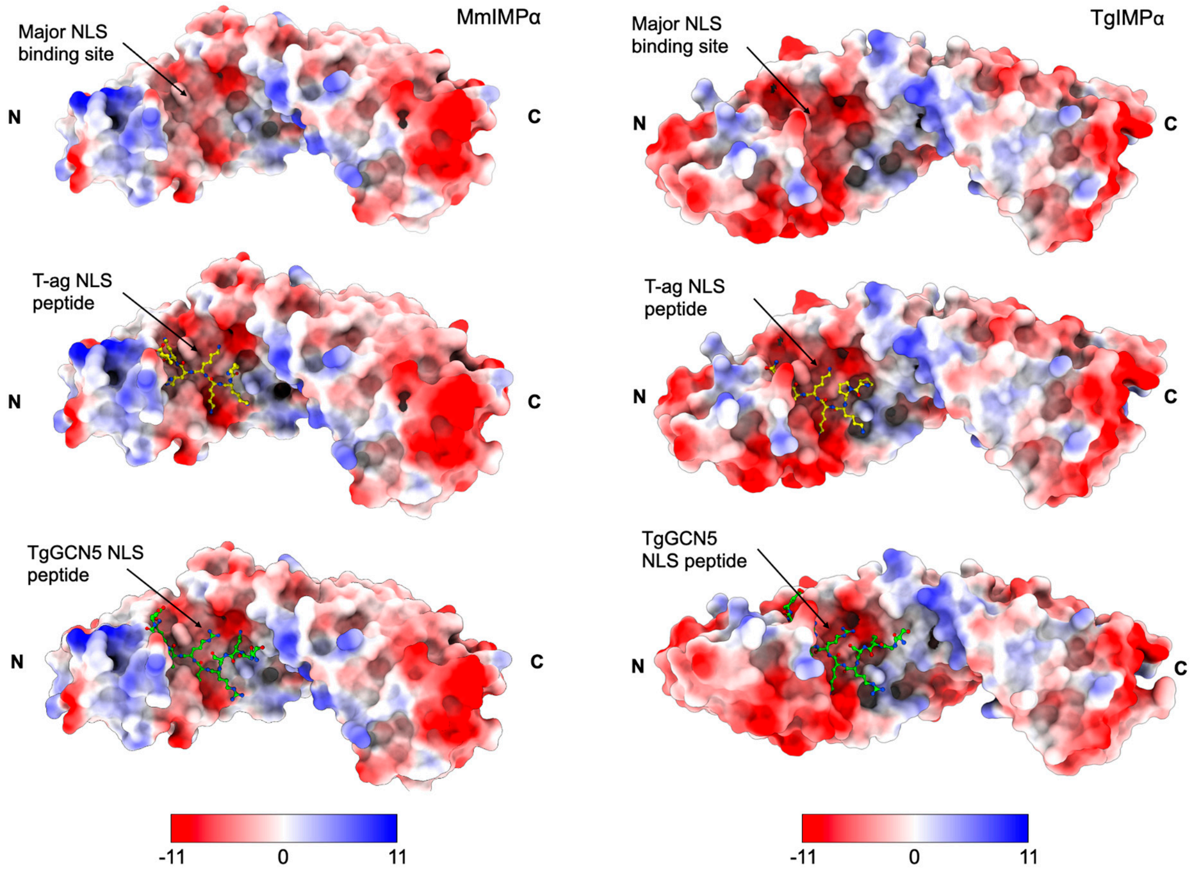

Figure S6. Electrostatic surface representation of the ARM repeats region of MmIMPα(72-497) and TgIMPα(80-507) highlights the acidic nature of the NLS binding groove, images generated using ChimeraX-1.10.1. Red and blue denote negatively and positively charged residue side chains respectively. Binding conformation of the T-ag NLS (yellow, stick-and-ball representation) and TgGCN5 NLS (green, stick-and-ball representation) in the major NLS binding groove is shown in the middle panel and lower panels respectively.

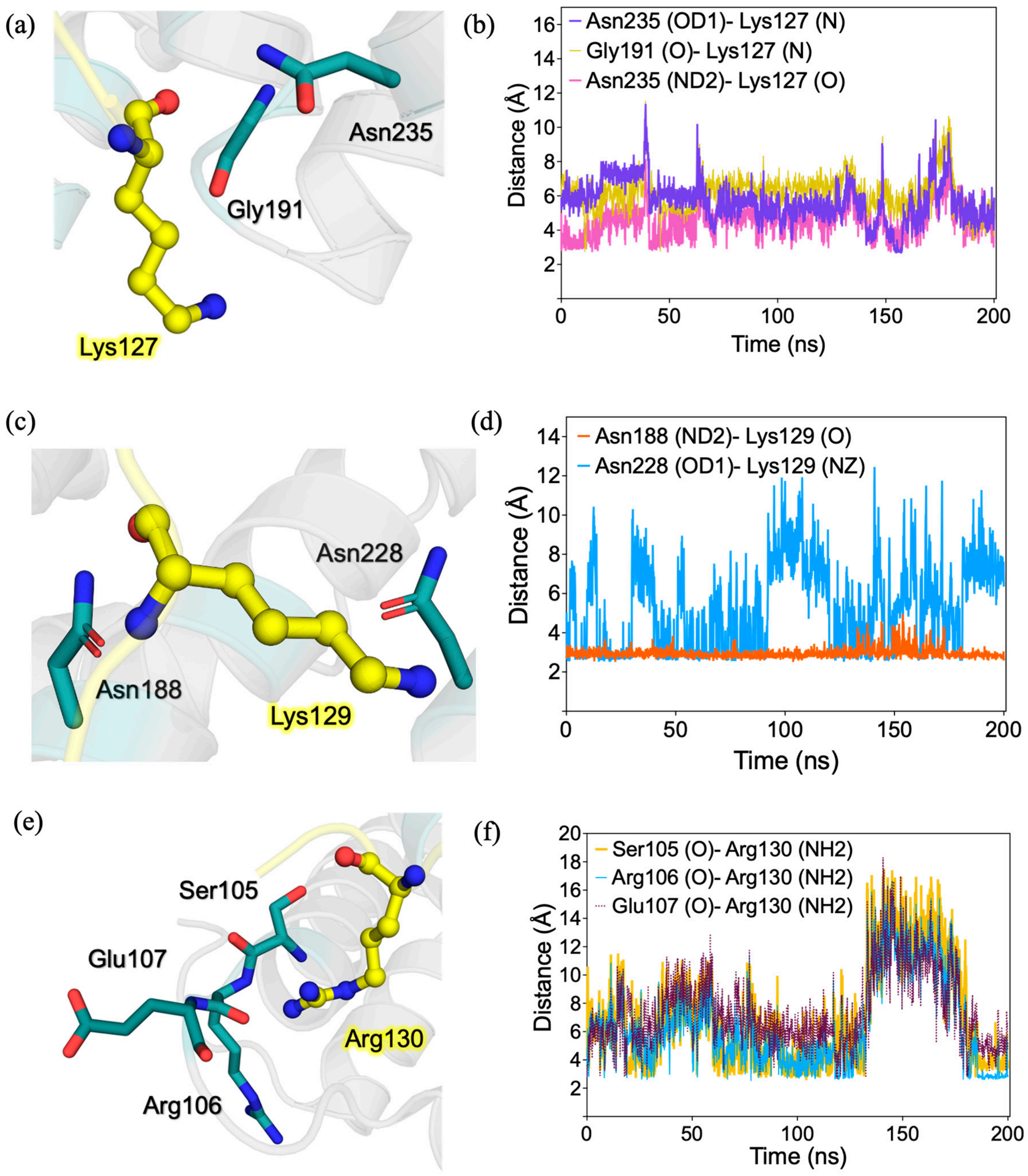

Figure S7. Interactions of MmIMPα(72-497) with residues Lys127, Lys129, and Arg130 of the T-ag-NLS as revealed by MD simulations, revealing stable polar interaction networks with conserved Asn residues and backbone oxygen atoms of MmIMPα. Ball and stick representation of interactions of MmIMPα(72-497) with Lys127 (a), Lys129 (c) and Arg130 (e) of the T-ag-NLS. RMSF plots show persistent polar interactions between Asn188 (d) and the backbone of Lys129 (middle), while the interactions with Lys127 (b) and Arg130 (f) appear to have variable interatomic distances over the 200-ns simulation trajectory implying they are more dynamic.

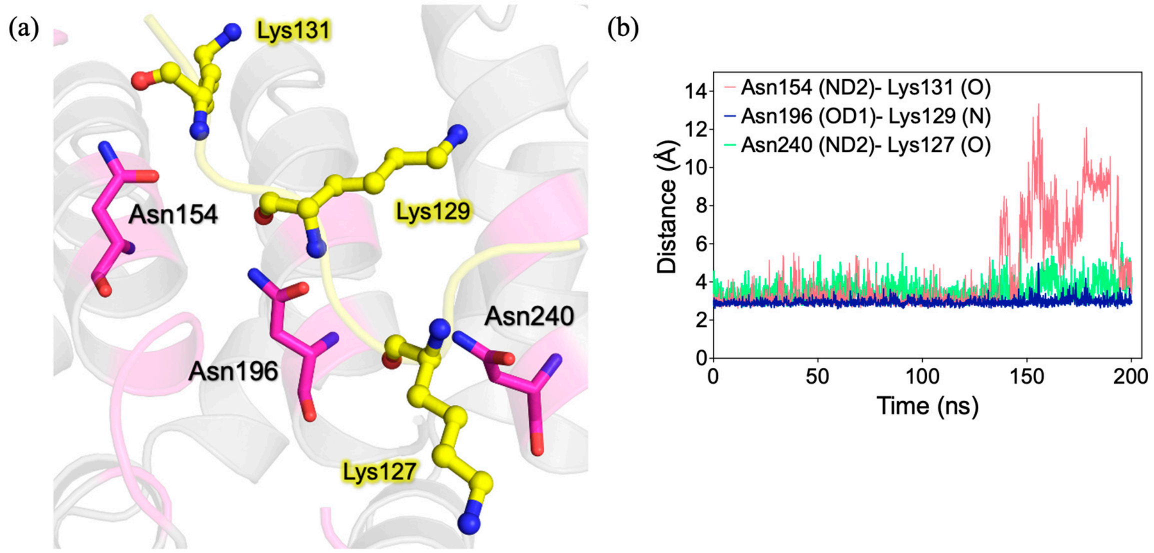

Figure S8. **MD simulations reveal Asn-mediated polar interactions between the T-ag NLS and TgIMPα(80-507).** (a) Ball-and-stick representation depicts polar contacts between T-ag NLS residues Lys127, Lys129, and Lys131 and TgIMPα residues Asn240, Asn196, and Asn154, respectively, within the major NLS-binding groove. (b) RMSF plots show persistent interactions with short interatomic distances between these residue pairs, except for the Asn154-Lys131 interaction, over the 200-ns simulation trajectory.

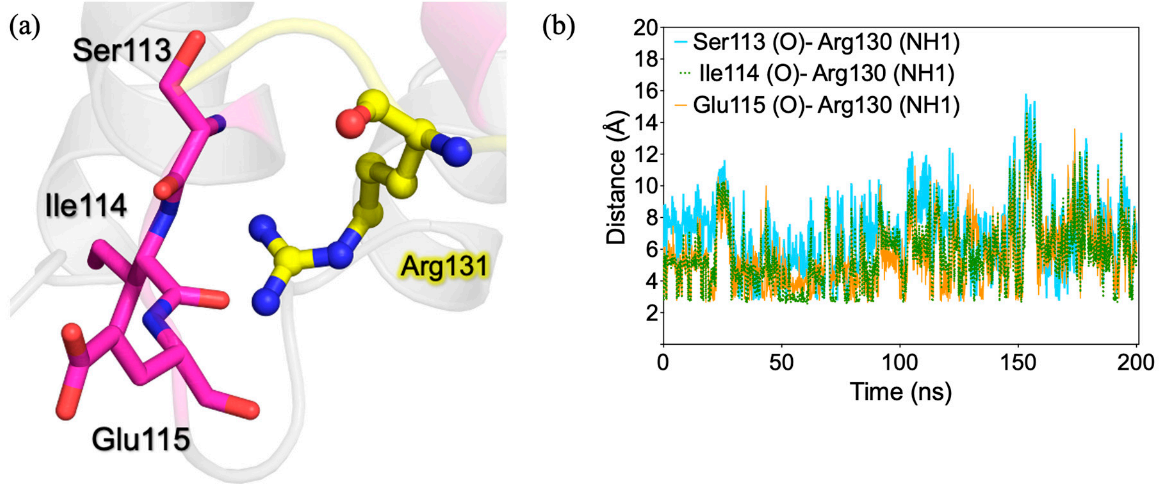

Figure S9. **MD simulations reveal peripheral interactions between Arg130 of the T-ag NLS and backbone carbonyl oxygens in ARM1 of TgIMPα(80-507).** (a) Ball-and-stick representation shows Arg130 (NH1) of the T-ag NLS interacting with the backbone carbonyl oxygens of Ser113, Ile114, and Glu115 in ARM1 of TgIMPα(80-507). (b) RMSF plots show variable interatomic distances between these residues over the 200-ns simulation trajectory, consistent with transient interactions.

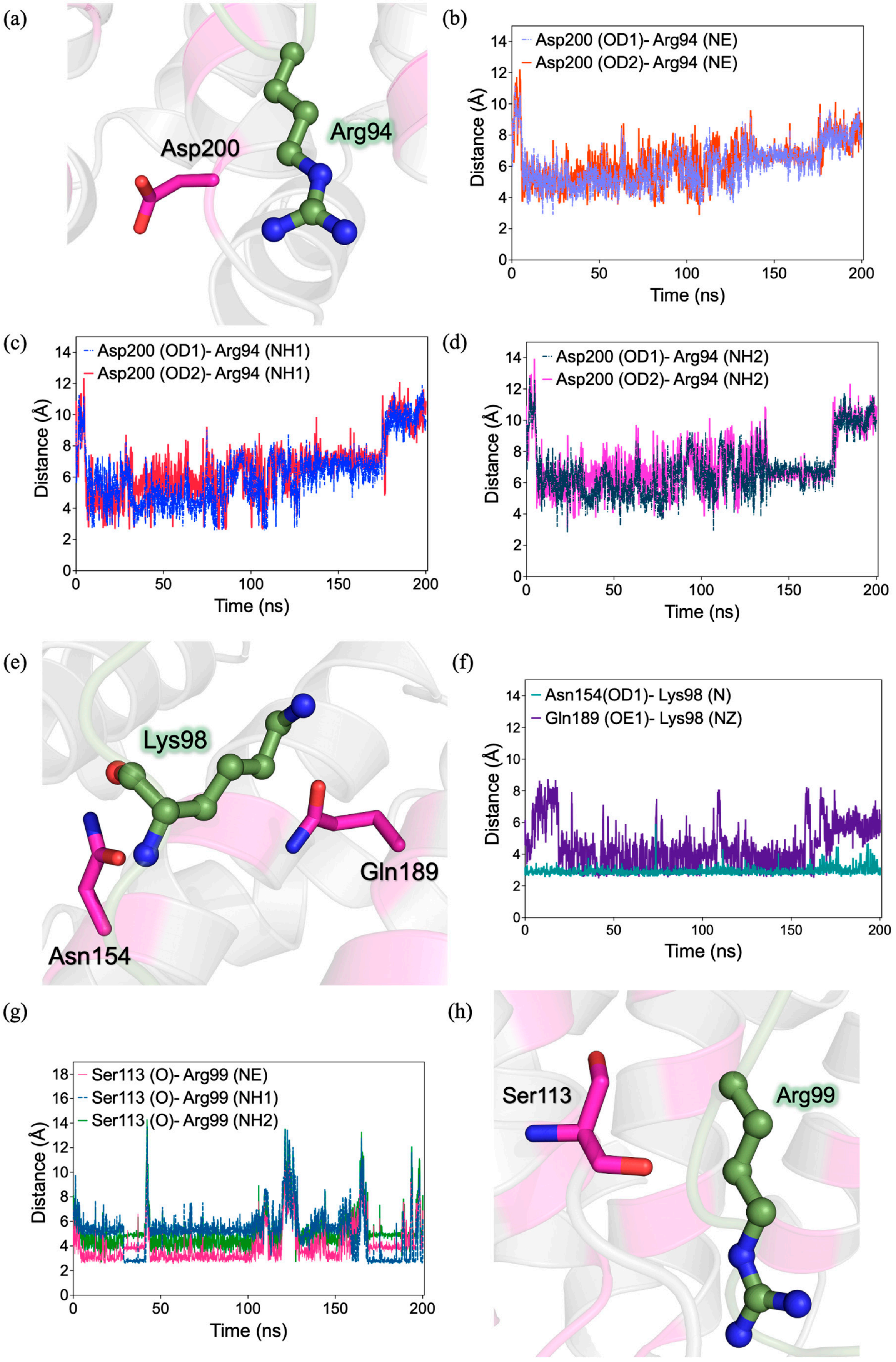
(a)
Asp200
Arg94
(b)
Asp200 (OD1)- Arg94 (NE)
Asp200 (OD2)- Arg94 (NE)
Distance (Å)
Time (ns)
(c)
Asp200 (OD1)- Arg94 (NH1)
Asp200 (OD2)- Arg94 (NH1)
Distance (Å)
Time (ns)
(d)
Asp200 (OD1)- Arg94 (NH2)
Asp200 (OD2)- Arg94 (NH2)
Distance (Å)
Time (ns)
(e)
Lys98
Gln189
Asn154
(f)
Asn154(OD1)- Lys98 (N)
Gln189 (OE1)- Lys98 (NZ)
Distance (Å)
Time (ns)
(g)
Ser113 (O)- Arg99 (NE)
Ser113 (O)- Arg99 (NH1)
Ser113 (O)- Arg99 (NH2)
Distance (Å)
Time (ns)
(h)
Ser113
Arg99

Figure S10. MD simulations identify key stabilizing interactions between residues Arg94 and Lys98, and peripheral interactions with Arg99 of the TgGCN5 NLS and binding groove residues of TgIMPα(80-507) over a 200-ns simulation trajectory. (a) Ball-and-stick representation illustrating the structural arrangement of Arg94 relative to Asp200 within the major NLS-binding groove. RMSF plots show variable interatomic distances between the NE (b), NH1 (c), and NH2 (d) atoms of Arg94 and the carboxylate oxygen atoms of Asp200. (e) Ball-and-stick representation depicting polar contacts between Lys98 and Asn154 and Gln189 of TgIMPα(80-507). (f) RMSF curves demonstrating persistent short interatomic distances with Asn154 and more dynamic interactions with Gln189. (g) RMSF curves revealing dynamic peripheral interactions involving all three nitrogen (NE, NH1 and NH2) atoms of Arg99 throughout the 200-ns simulation. (h) Ball-and-stick representation illustrating the structural configuration of Arg99 relative to Ser113 of TgIMPα(80-507).

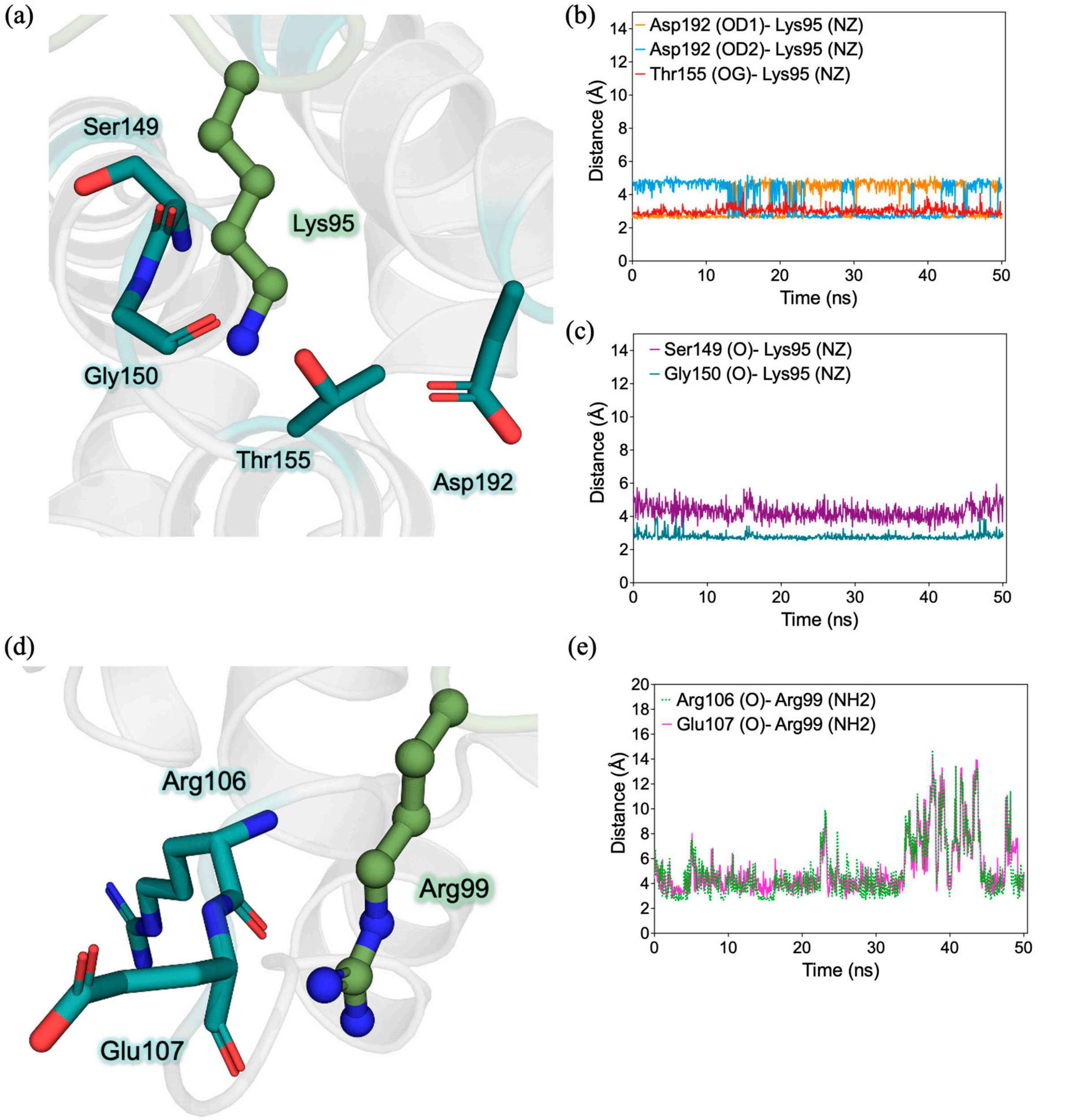

Figure S11. MD simulations reveal polar and peripheral interactions of the TgGCN5 NLS with MmIMPα(72-497). (a) Ball-and-stick representation shows Lys95 of the TgGCN5 NLS forming polar contacts with Thr155, Ser149, and Gly150, and a salt-bridge interaction with Asp192 within the major NLS-binding groove of MmIMPα(72-497). (b) RMSF plots show short interatomic distances between Lys95 and the side chain atoms of Asp192 and Thr155. (c)RMSF plots with the backbone carbonyl oxygen atoms of Ser149 and Gly150. (d) Ball-and-stick representation shows the structural configuration of Arg99 relative to the peripheral residues Arg106 and Glu107 in the ARM1 region. (e) RMSF plots show variable interatomic distances between the NH2 atom of Arg99 and the backbone carbonyl oxygen atoms of Arg106 and Glu107 over the 200-ns simulation trajectory.

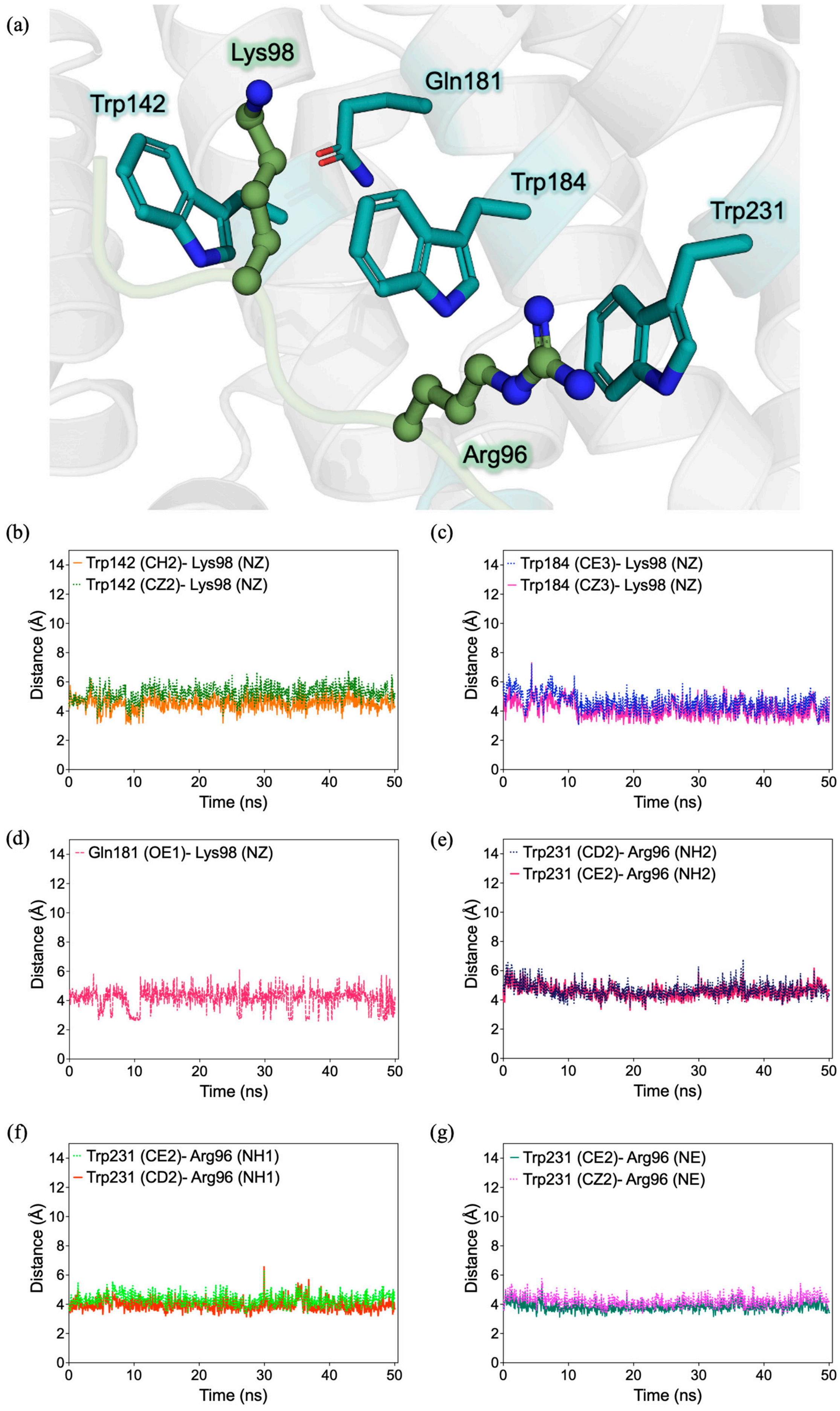
(a)
Lys98
Gln181
Trp142
Trp184
Trp231
Arg96
(b)
Trp142 (CH2)- Lys98 (NZ)
Trp142 (CZ2)- Lys98 (NZ)
Distance (Å)
Time (ns)
(c)
Trp184 (CE3)- Lys98 (NZ)
Trp184 (CZ3)- Lys98 (NZ)
Distance (Å)
Time (ns)
(d)
Gln181 (OE1)- Lys98 (NZ)
Distance (Å)
Time (ns)
(e)
Trp231 (CD2)- Arg96 (NH2)
Trp231 (CE2)- Arg96 (NH2)
Distance (Å)
Time (ns)
(f)
Trp231 (CE2)- Arg96 (NH1)
Trp231 (CD2)- Arg96 (NH1)
Distance (Å)
Time (ns)
(g)
Trp231 (CE2)- Arg96 (NE)
Trp231 (CZ2)- Arg96 (NE)
Distance (Å)
Time (ns)

Figure S12. MD simulations highlight cation-π interactions between the TgGCN5 NLS and MmIMPα(72-497). (a) Ball-and-stick representation illustrates a stacking arrangement in which Lys98 of the TgGCN5 NLS engages Trp142, Gln181, and Trp184, while Arg96 is positioned between Trp184 and Trp231 within the major NLS-binding groove of MmIMPα(72-497). RMSF plots show short interatomic distances between (b) Lys98 and Trp142, (c) Trp184, and (d)Gln181, as well as variable interatomic distances involving the (e) NH2, (f) NH1, and (g) NE atoms of Arg96 with surrounding aromatic residues over the 200-ns simulation trajectory.
